# Continuation vs Switching Direct Oral Anticoagulant Therapy After Breakthrough Stroke

**DOI:** 10.1001/jamanetworkopen.2026.9584

**Published:** 2026-04-28

**Authors:** Lucio D’Anna, Francesca Gabriele, Raffaele Ornello, Andrea Zini, Matteo Paolucci, Stefano Forlivesi, Ludovica Migliaccio, Maria Maddalena Viola, Angelo Cascio Rizzo, Maria Sessa, Ghil Schwarz, Rachele Tortorella, Gabriele Prandin, Soma Banerjee, Gurav Desai, Leonardo Pantoni, Francesco Mele, Giuseppe Scopelliti, Ilaria Cova, Mariarosaria Valente, Domenico Maisano, Maria Rosaria Bagnato, Giovanni Di Mauro, Francesca Bernocchi, Martina Gaia Di Donna, Barbara Casolla, Marie-Helene Mahagne, Marie-Eve Amoretti, Lucille Morgan, Laura González, Ricardo Rigual, Blanca Fuentes, Carlos Hervás, Paolo Candelaresi, Vincenzo Andreone, Antonio De Mase, Emanuele Spina, Diana Aguiar de Sousa, Mariana Almudi Souza, Alberto Fior, Miguel Serôdio, Pietro Caliandro, Aurelia Zauli, Giuseppe Reale, Ahmed Abdelalim, Sandra Ahmed, Samah Ali Ismail, Liqun Zhang, Tara Latimer, Muhammad Elboghday, Ahmed El Bassiouny, Tamer Roushdy, Hossam Shokri, Federica Ferrari, Nicola Davide Loizzo, Federico Mazzacane, Maria Guarion, Valentina Barone, Paola Forti, Giuseppe Rinaldi, Marco Rossi, Vincenzo Laterza, Giovanni Frisullo, Pier Andrea Rizzo, Aldobrando Broccolini, Marina Mannino, Valeria Terruso, Marcella Caggiula, Simona Scalise, Ana Catarina Fonseca, Bernardo Antunes, Hrvoje Budincevic, Petra Crnac, Giovanna Viticchi, Mauro Silvestrini, Lorenzo Barba, Viktoria Musienko, Piergiorgio Lochner, Benjamin Landau, Sandeep Buddha, Roumeisa Khalil, Maria Grazia Piscaglia, Elena Minguzzi, Marialuisa Zedde, Ahmed Nasreldein, Luisa Vinciguerra, Luis Costa, Ahmed ElsaidElsayed, Mona Al Banna, Laura Tudisco, Maria Giulia Mosconi, Giovanni Merlino, Federico De Santis, Simona Sacco, Matteo Foschi

**Affiliations:** 1 Department of Stroke and Neuroscience, Charing Cross Hospital, Imperial College London NHS (National Health Service) Healthcare Trust, London, United Kingdom; 2 Department of Brain Sciences, Imperial College London, London, United Kingdom; 3 Department of Biotechnological and Applied Clinical Sciences, University of L’Aquila, L’Aquila, Italy; 4 Istituto di Ricovero e Cura a Carattere Scientifico (IRCCS) Istituto delle Scienze Neurologiche di Bologna, Department of Neurology and Stroke Center, Maggiore Hospital, Bologna, Italy; 5 Department of Neurology and Stroke Unit, Azienda Socio Sanitaria Territoriale (ASST) Grande Ospedale Metropolitano Niguarda, Milan, Italy; 6 Clinical Unit of Neurology, Department of Medicine, Surgery and Health Sciences, University Hospital and Health Services of Trieste, Azienda Sanitaria Universitaria Giuliano Isontina, University of Trieste, Trieste, Italy; 7 Neuroscience Research Center, Department of Biomedical and Clinical Sciences, University of Milan, Milan, Italy; 8 Department of Neurorehabilitation Sciences, Casa di Cura Igea, Milan, Italy; 9 Neurology Unit, University Hospital Luigi Sacco, Milan, Italy; 10 Clinical Neurology, Department of Medicine, University of Udine, Italy; 11 L’Unità Operativa Complessa (UOC) Stroke Unit e Neurologia, Ospedale Fabrizio Spaziani, Frosinone, Italy; 12 Stroke Unit, Centre Hospitalier Universitaire Pasteur 2, Université Cote d’Azur, Unité Mixte de Recherche Clinique Côte d’Azur, Unité de Recherche sur le Syndrome Radiologique Isolé, Nice, France; 13 Stroke Center and Department of Neurology, Hospital La Paz Institute for Health Research, La Paz University Hospital–Universidad Autónoma de Madrid, Madrid, Spain; 14 UOC Neurologia e Stroke Unit, Azienda Ospedaliera di Rilievo Nazionale Cardarelli, Napoli, Italy; 15 Stroke Center, Department of Neurosciences, Centro Hospitalar Universitário Lisboa Central–Unidade Local de Saúde (ULS) São José, and Faculdade de Medicina, Universidade de Lisboa, Lisbon, Portugal; 16 Stroke Center, Department of Neurosciences, Centro Hospitalar Universitário Lisboa Central–ULS São José, Lisbon, Portugal; 17 Department of Neuroscience, Catholic University of the Sacred Hearth, Rome, Italy; 18 UOC Neurology, Department of Neuroscience, Sensory Organs, and Thorax, Fondazione Policlinico Universitario A. Gemelli IRCCS, Rome, Italy; 19 UOC Neuroriabilitazione ad Alta Intensità, Fondazione Policlinico Universitario A. Gemelli IRCCS, Rome, Italy; 20 Cairo University Stroke Center, Department of Neurology, Faculty of Medicine, Cairo University, Cairo, Egypt; 21 Department of Neurology, St George’s University Hospital, London, United Kingdom; 22 Neurology Department, Faculty of Medicine, Ain Shams University, Cairo, Egypt; 23 Department of Brain and Behavioral Sciences, University of Pavia, Pavia, Italy; 24 Department of Emergency Neurology and Stroke Unit, IRCCS Mondino Foundation, Pavia, Italy; 25 IRCCS Istituto delle Scienze Neurologiche di Bologna, Bologna, Italy; 26 Department of Medical and Surgical Sciences, University of Bologna, IRCCS Azienda Ospedaliero–Universitaria di Bologna, Bologna, Italy; 27 S.C. Neurologia, Ospedale Di Venere, Bari, Italy; 28 Emergency Neurology, Department of Neuroscience, Sensory Organs, and Thorax, Policlinico Universitario Agostino Gemelli, IRCCS Rome, Rome, Italy; 29 Catholic University of the Sacred Heart–Fondazione Policlinico Agostino Gemelli IRCCS, Rome, Italy; 30 Stroke Unit, Department of Neuroscience, Sensory Organs, and Thorax, Policlinico Universitario Agostino Gemelli, IRCCS Rome, Rome, Italy; 31 UOC Neurologia e Stroke Unit, Azienda Ospedaliera Ospedali Riuniti Villa Sofia-Cervello, Palermo, Italy; 32 UOC Neurologia e Stroke Unit, Po Vito Fazzi, Lecce, Italy; 33 Neurology Department, Hospital de Santa Maria, Faculdade de Medicina, University of Lisbon, Portugal; 34 Department of Neurology, Hospital de Santa Maria, Lisbon, Portugal; 35 Department of Neurology, Sveti Duh University Hospital, Zagreb, Croatia; 36 Department of Neurology and Neurosurgery, Faculty of Medicine, J.J. Strossmayer University of Osijek, Osijek, Croatia; 37 Department of Psychiatry and Neurology, Faculty of Dental Medicine and Health, J.J. Strossmayer University of Osijek, Osijek, Croatia; 38 Neurological Clinic, Marche Polytechnic University, Ancona, Italy; 39 Department of Neurology, Martin-Luther-University Halle-Wittenberg, Halle, Germany; 40 Department of Neurology, Saarland University Medical Center, Homburg, Germany; 41 Department of Stroke Medicine, Southmead Hospital, North Bristol NHS Trust, Bristol, United Kingdom; 42 Department of Neurosciences, Stroke Unit–Neurology Unit, S. Maria delle Croci Hospital, Azienda Unità Sanitaria Locale della Romagna, Ravenna, Italy; 43 Neurology Unit, Stroke Unit, Azienda Unità Sanitaria Locale–IRCCS di Reggio Emilia, Italy; 44 Department of Neurology, Assiut University, Assiut, Egypt; 45 Department of Neurology and Stroke Unit, ASST Crema Hospital, Crema, Italy; 46 Department of Neurology, Local Health Unit of Alto Minho, Viana do Castelo, Portugal; 47 Neurology and Neurointervention Department, Kobry El Koba Medical Complex, Cairo, Egypt; 48 Department of Neurology, National Neuroscience Institute, King Fahad Medical City, Riyadh, Saudi Arabia; 49 Stroke Unit, Careggi University Hospital, Florence, Italy; 50 Department of Internal and Cardiovascular Medicine, Santa Maria della Misericordia Hospital, Perugia, Italy; 51 Struttura Operativa Semplice Dipartimentale Stroke Unit, Udine University Hospital, Udine, Italy

## Abstract

**Importance:**

Management after an ischemic stroke occurring despite direct oral anticoagulant (DOAC) therapy for atrial fibrillation (AF) varies widely. Switching anticoagulation is common in clinical practice, although evidence supporting this strategy is limited.

**Objective:**

To evaluate whether continuation of treatment with the same DOAC was noninferior to switching oral anticoagulant therapy with respect to 90-day clinical outcomes.

**Design, Setting, and Participants:**

This multicenter registry-based cohort study with an emulated target trial design included consecutive adult patients with AF who experienced a breakthrough ischemic stroke while receiving uninterrupted DOAC therapy and resumed anticoagulation therapy thereafter. Patients were enrolled between February 2020 and February 2025, across 35 stroke centers in 9 countries in Europe and North Africa, with a standardized 90-day follow-up. The dataset was locked on September 1, 2025. A noninferiority comparison of switching vs continuation strategies was performed. Baseline confounding was addressed using inverse probability of treatment weighting (IPTW). The primary noninferiority margin was an absolute risk difference of 3.0 percentage points in 90-day net clinical benefit.

**Exposure:**

The intervention group switched to treatment with a different DOAC or vitamin K antagonist; the comparator group continued therapy with the prestroke DOAC.

**Main Outcomes and Measures:**

The primary outcome was 90-day net clinical benefit, defined as recurrent ischemic stroke and moderate to severe bleeding. Secondary outcomes included recurrent ischemic events, symptomatic intracerebral hemorrhage, moderate to severe extracranial bleeding, all-cause mortality, and vascular death.

**Results:**

Among 1006 patients included in the analysis (median age, 80.4 [IQR, 73.4-85.4] years; 503 female [50.0%] and 503 [50.0%] male), 463 (46.0%) continued the same DOAC therapy and 543 (54.0%) switched therapy. After IPTW adjustment, the 90-day net clinical benefit was 4.9% with switching and 5.1% with continuation, corresponding to a risk difference of −0.3 percentage points (90% CI, −2.7 to 2.1 percentage points), meeting the prespecified noninferiority criterion. For recurrent ischemic events and bleeding outcomes, the absolute differences were within the predefined noninferiority margins. Noninferiority was not demonstrated for all-cause or vascular mortality.

**Conclusions and Relevance:**

In patients with breakthrough ischemic stroke during DOAC therapy, switching anticoagulation treatment was not associated with clinically meaningful short-term benefit compared with continuation. These findings suggest that switching does not provide additional benefit compared with continuing treatment with the same DOAC. Randomized clinical trials are needed to identify strategies to improve secondary prevention after a breakthrough ischemic stroke.

## Key Points

**Question:**

Among patients with atrial fibrillation experiencing a breakthrough ischemic stroke while receiving direct oral anticoagulant (DOAC) therapy, is switching to another oral anticoagulant associated with 90-day outcomes that are not meaningfully worse than continuing treatment with the same DOAC?

**Findings:**

In this cohort study including 1006 patients, 463 continued the same DOAC therapy and 543 switched to a different DOAC. Switching to a different DOAC was noninferior to continuing the same DOAC therapy in terms of a 90-day net clinical benefit.

**Meaning:**

These findings suggest that switching DOACs after a breakthrough stroke may not be associated with clinically meaningful benefits in short-term outcomes.

## Teaser

This cohort study evaluates whether continuation of treatment with a direct oral anticoagulant (DOAC) was noninferior to switching DOAC therapy with respect to 90-day clinical outcomes among patients with atrial fibrillation and breakthrough stroke.

## Introduction

Direct oral anticoagulants (DOACs) have become the preferred treatment option over vitamin K antagonists (VKAs) for stroke prevention in patients with atrial fibrillation (AF).^1,2^ However, despite optimal DOAC therapy, breakthrough ischemic strokes occur in approximately 1% of DOAC-treated patients each year,^3^ and the risk of recurrence after such events remains high, ranging from 5% to 9% annually.^1^ Potential causes might include poor adherence to therapy or underdosing, as well as other competing causes of stroke,^4^ such as small vessel disease or large artery atherosclerosis. Notably, breakthrough strokes may also occur even in the context of verified adherence and guideline-appropriate DOAC dosing. In those cases, the optimal secondary prevention strategy has not yet been defined.^5^ Nonetheless, switching oral anticoagulants is a common clinical practice in this context.^6^ The motivations for switching vary across studies, but the most frequently cited rationale is the expectation of improved efficacy when using an agent with a different pharmacologic mechanism of action.^7^ However, despite the absence of randomized evidence and without a clear biological rationale supporting a benefit from switching, this strategy remains widespread in routine care, often driven by the perception of treatment failure rather than evidence-based decision making.^6^ To formally address this question, we emulated a target trial to minimize bias inherent in observational datasets.^8,9^ We evaluated whether continuation of the same DOAC therapy was noninferior to switching oral anticoagulant therapy with respect to 90-day clinical outcomes, using prespecified clinically acceptable margins.

## Methods

### ASPERA Project

The Advancing Knowledge in Ischemic Stroke Patients on Oral Anticoagulants (ASPERA) study^10^ is a large, multicenter, observational, clinical investigation coordinated by the University of L’Aquila, L’Aquila, Italy. It consists of 2 complementary arms: a retrospective cohort (ASPERA-R) that is designed to collect baseline characteristics and 90-day outcomes in patients with AF who experienced a breakthrough ischemic stroke (ie, an ischemic stroke occurring despite ongoing, continuous anticoagulation) and a prospective arm (ASPERA-P) that is aimed at evaluating long-term outcomes as long as 5 years, which is currently ongoing. We report herein data from the ASPERA-R arm. The ASPERA-R study involved 35 stroke centers across 9 countries in Europe and North Africa (eAppendix 1 in Supplement 1). Participating centers were predominantly located in tertiary care hospitals with comprehensive stroke centers with established stroke unit care and structured follow-up pathways (listed in eAppendix 2 in Supplement 1). All centers participating in the ASPERA study were required to routinely apply comprehensive diagnostic testing for stroke etiologic assessment as part of standard clinical practice. Likewise, participation in the ASPERA-R study was restricted to centers with an established and routinely implemented 90-day follow-up pathway for all patients with stroke, including standardized in-person outcome assessment and structured telephone contact for patients unable to attend in-person consultations. In addition, participating centers followed predefined case identification procedures to ensure consecutive inclusion of eligible patients and to minimize the risk of selection bias. Between February 2020 and February 2025, the ASPERA-R registry included consecutive adult patients (aged >18 years) with a documented diagnosis of AF who experienced an ischemic stroke while receiving ongoing oral anticoagulation therapy. Further details of the ASPERA-R have been defined elsewhere^11^ and can be found in the eMethods in Supplement 1. The final dataset for this analysis was locked on September 1, 2025.

### Ethics and Reporting

The ASPERA study received approval from the territorial ethics committee of the Abruzzo region in February 2025. Informed consent for inclusion in the ASPERA-R study was not required in most participating countries, where ethics committees granted a waiver because patient identification, baseline characteristics, and exposure ascertainment were based on retrospective review of medical records. In jurisdictions where consent is mandated even for retrospective studies (eg, Italy), written informed consent was obtained from patients or from a legally authorized representative; for deceased or unreachable patients, the ethics committee approved a waiver of consent. The study followed the Strengthening the Reporting of Observational Studies in Epidemiology (STROBE) reporting guideline. A synopsis of the trial protocol is provided in eAppendix 3 in Supplement 1.

### Emulated Target Trial Design, Population, and Methods

In the setting of ASPERA-R, target trial emulation was applied as a structured methodologic framework to prespecify eligibility criteria, treatment strategies, definition of time zero, follow-up procedures, estimand, and analytic approach, thereby aligning the observational analysis with the design principles of a randomized clinical trial. The emulated target trial compared 2 poststroke anticoagulation strategies in patients with AF who experienced a breakthrough ischemic stroke while receiving DOAC therapy: switching to an alternative oral anticoagulant (including another DOAC or a VKA) vs continuation of the same DOAC. A noninferiority framework was adopted because switching anticoagulant therapy in this context is already common clinical practice, despite the absence of robust evidence demonstrating superiority. The primary inferential objective was therefore to determine whether continuation of the same DOAC was not clinically inferior to switching therapy with respect to prespecified 90-day outcomes. Switching DOAC therapy was treated as the intervention strategy, and continuation of the same DOAC as the reference strategy throughout the analyses. Eligible patients for the purpose of this analysis, within the ASPERA-R registry population, were adults with AF who experienced a breakthrough ischemic stroke while receiving uninterrupted DOAC therapy, defined as documented intake during the 7 days preceding the index event with the last dose administered within 48 hours before stroke onset. Patients were required to resume oral anticoagulation after the index event with either continuation of the same DOAC or switching to an alternative oral anticoagulant. The exclusion criteria for the emulated trial were (1) presence of a mechanical prosthetic heart valve; (2) receipt of treatment with heparin, low-molecular-weight heparin, or VKA prior to the index event; (3) failure to resume any oral anticoagulant therapy (DOAC or VKA) after the index ischemic stroke; (4) use of heparin or low-molecular-weight heparin as the poststroke anticoagulation strategy instead of DOAC; and (5) left atrial appendage occlusion.

### Emulated Target Trial Outcomes

All primary and secondary outcomes were ascertained by study investigators through systematic review of 90-day follow-up visits. The primary outcome was net clinical benefit at 90 days, defined as the composite of recurrent ischemic stroke and moderate to severe bleeding. Secondary outcomes included 90-day occurrence of a new ischemic stroke after the index breakthrough event, defined as a new neurologic deficit with a corresponding acute ischemic lesion on noncontrast computed tomography or magnetic resonance imaging of the brain, including cases with symptom resolution within 24 hours (transient ischemic attacks) and the 90-day occurrence of any new ischemic event (stroke or systemic embolism). Safety outcomes included 90-day moderate to severe bleeding, symptomatic intracerebral hemorrhage (ICH), all-cause death, and vascular death (any death attributable to cardiovascular or cerebrovascular causes, including fatal ischemic or hemorrhagic stroke, sudden cardiac death, myocardial infarction, or other vascular events). Bleeding severity was categorized according to the GUSTO (Global Utilization of Streptokinase and Tissue Plasminogen Activator for Occluded Coronary Arteries) trial classification.^12^ Specifically, bleeding events were considered moderate to severe if they resulted in fatal hemorrhage or required blood or fluid replacement, inotropic support, or surgical intervention, regardless of hemodynamic compromise. Symptomatic ICH was defined as any ICH associated with neurologic worsening of 4 or more points on the National Institutes of Health Stroke Scale score, not attributable to other causes.^13^ All primary and secondary outcomes were ascertained by investigators through systematic review of 90-day follow-up assessments and medical records, using predefined operational definitions. The noninferiority framework and prespecified margins used for outcome comparisons are detailed below.

### Statistical Analysis

Baseline characteristics were summarized as medians with IQRs for continuous variables and as counts with percentages for categorical variables. Between-group differences for patients who changed therapy vs those who continued the same DOAC were assessed using the Mann-Whitney test or χ^2^ test, as appropriate. Covariate balance between the 2 groups was evaluated using standardized mean differences (SMD), with values less than 0.1 indicating adequate balance.^14^

To emulate a target trial framework, inverse probability of treatment weighting (IPTW) based on propensity scores was used to adjust for baseline imbalances between groups. The primary analysis targeted the mean treatment effect, estimating the marginal effect of switching vs continuation in the overall target population. The propensity model included demographic, clinical, imaging, and treatment variables identified a priori as potential confounders (eTable 1 in Supplement 1). Stabilized weights were applied to reduce variance, and postweighting balance was verified using weighted SMDs and visual inspection with Love plots.

Weighted logistic regression models with robust standard errors were then used to estimate risk differences (RDs) and risk ratios (RRs) with corresponding 90% CIs. Survey-weighted generalized linear models were used, specifying stabilized IPTW weights as probability weights, to obtain valid point estimates and robust standard errors for RDs and RRs in the weighted pseudopopulation.

The trial was emulated using a noninferiority framework, reflecting the clinical equipoise that switching anticoagulant therapy after breakthrough ischemic stroke is not clearly superior to continuation, while potentially introducing additional bleeding risk. Therefore, the primary hypothesis was that continuing the same DOAC therapy would be noninferior to switching DOAC therapy with respect to the 90-day net clinical benefit outcome. Noninferiority was evaluated primarily on the absolute RD scale, with supportive assessment on the RR scale. In accordance with European Medicines Agency guidance, noninferiority margins were prespecified to represent the maximum clinically acceptable difference between strategies and were justified based on prior evidence rather than observed data.^15^

Before study initiation, a sample size estimation was performed for the primary noninferiority hypothesis. Assuming a 5% event rate in the reference group and a noninferiority margin of 3% on the absolute RD scale, approximately 800 patients (1-sided α = .05; 80% power; balanced allocation) were required. The anticipated registry size exceeded this threshold. Throughout the report, switching oral anticoagulant therapy was considered the intervention strategy, whereas continuation of the same DOAC therapy was considered the reference strategy. RDs are therefore expressed as switching minus continuation strategies. For the primary composite end point, an RD margin of 3.0 percentage points (pp) was selected as the largest tolerable excess absolute risk that would still justify avoiding unnecessary treatment modification. More stringent margins were applied for safety outcomes (0.5 pp for symptomatic ICH at 90 days; 1.0 pp for moderate to severe extracranial bleeding at 90 days), consistent with regulatory recommendations that safety outcomes warrant tighter clinical acceptability thresholds.^16^ Slightly wider margins were considered acceptable for recurrent ischemic events and all-cause mortality, where baseline risks are higher and small absolute differences are less likely to be clinically meaningful. The selection of noninferiority margin was also based on magnitudes of commonly observed and clinically meaningful relative and absolute risk reductions in a wide range of secondary stroke preventive trials.^17^

In accordance with standard noninferiority methodology and regulatory guidance from the European Medicine Agency^18^ and the International Council of Harmonisation of Technical Requirements for Pharmaceutical for Human Use (ICH E9 guideline),^19^ treatment effects were estimated using 2-sided 90% CIs, corresponding to a 1-sided α level of .05. Noninferiority was concluded when the upper bound of the 90% CI for the RD did not exceed the prespecified margin. Noninferiority margins were defined a priori to represent the maximum clinically acceptable difference between strategies and were justified based on prior evidence rather than observed data. Supportive analyses on the RR scale used noninferiority margins derived from the prespecified RD margins to ensure clinical consistency across effect measures, rather than applying arbitrary or uniform relative thresholds. Sensitivity analyses included IPTW trimming (1st-99th and 5th-95th percentiles), overlap weighting targeting the mean treatment effect in the overlap population, which emphasizes patients with similar propensity scores and improves covariate overlap, augmented IPTW with 200 bootstrap replications, an unweighted covariate-adjusted regression, and a per-protocol analysis restricted to patients who resumed anticoagulation therapy within 10 days of the index stroke. As an additional prespecified sensitivity analysis, we repeated the primary noninferiority analysis after excluding patients with a competing stroke etiology. IPTWs previously estimated in the main analysis were retained, and weighted survey regression models were refitted in the restricted cohort to obtain RDs and RRs with 90% CIs for the net clinical benefit outcome. Additional exploratory comparisons evaluated specific anticoagulation strategies (eg, switching to another DOAC subtype or to a VKA vs continuing the same DOAC). All analyses were conducted using 2-sided tests with α = .05 indicating statistical significance in R, version 4.5 (R Project for Statistical Computing), using the WeightIt, survey, and marginal effects packages.

## Results

**Figure 1.**
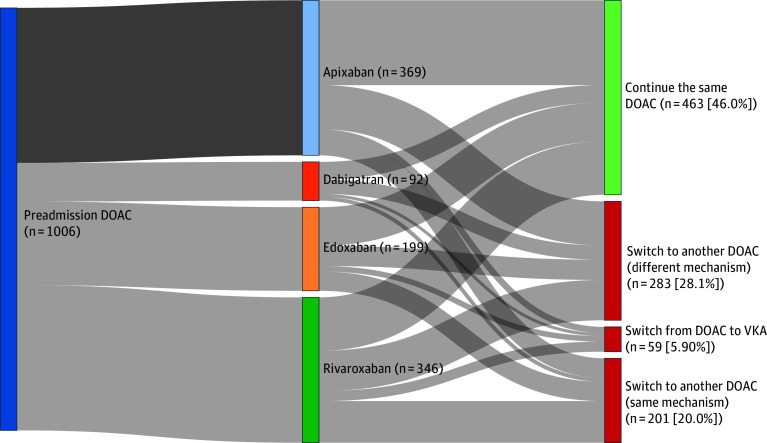
Sankey Diagram of Prestroke Direct Oral Anticoagulation (DOAC) Agent and Poststroke Anticoagulation Strategy The diagram represents the flow of anticoagulation management strategies in patients with atrial fibrillation who experienced an ischemic stroke while receiving a DOAC (N = 1006). Width of the connecting bands is proportional to the number of patients transitioning between categories. VKA indicates vitamin K antagonist.

Overall, the target trial cohort included 1006 participants (eFigure 1 in Supplement 1) with a median age of 80.4 (IQR, 73.4-85.4) years; 503 participants (50.0%) were female and 503 (50.0%) were male. The median National Institutes of Health Stroke Scale score was 9 (IQR, 4-17) (scores ranged from 0-42, with higher scores indicating greater severity of stroke) (Table). Of these, 463 patients (46.0%) continued the same DOAC therapy while 543 (54.0%) changed DOAC therapy. Before weighting, most baseline covariates showed small SMDs between the 2 groups, generally below the conventional threshold of 0.10, indicating limited imbalance between groups. After applying IPTW, all weighted SMDs were substantially reduced and fell below 0.10, indicating adequate covariate balance between the continuation and switching groups (Table and eTable 1 and eFigures 2 to 3 in Supplement 1). Strategy definitions, including continuation of the same DOAC therapy, switching to another DOAC with the same or different mechanism, and switching from a DOAC to VKA, are presented in Figure 1.

**Table.** Patient Characteristics

Characteristic	Study group, No. (%)		
	Overall (N = 1006)	DOAC continuation (n = 463)	DOAC switching (n = 543)
**Demographic**			
Age, median, (IQR), y	80.4 (73.4-85.4)	80.0 (72.3-84.9)	80.6 (74.2-85.8)
Sex			
Female	503 (50.0)	240 (51.8)	263 (48.4)
Male	503 (50.0)	223 (48.2)	280 (51.6)
Race and ethnicity			
Asian	5 (0.5)	1 (0.2)	4 (0.7)
Black	19 (1.9)	14 (3.0)	5 (0.9)
Hispanic White	65 (6.5)	37 (8.0)	28 (5.2)
Non-Hispanic White	791 (78.6)	329 (71.1)	462 (85.1)
Other^a^	126 (12.5)	82 (17.7)	44 (8.1)
**Risk factors**			
Cigarette smoking	94 (9.3)	46 (9.9)	48 (8.8)
Hypertension	833 (82.8)	379 (81.9)	454 (83.6)
Dyslipidemia	522 (51.9)	248 (53.6)	274 (50.5)
Diabetes	276 (27.4)	130 (28.1)	146 (26.9)
Ischemic heart disease	229 (22.8)	113 (24.4)	116 (21.4)
Cancer	136 (13.5)	58 (12.5)	78 (14.4)
Previous TIA or stroke	270 (26.8)	130 (28.1)	140 (25.8)
Previous ICH	17 (1.7)	10 (2.2)	7 (1.3)
Chronic congestive heart failure	161 (16.0)	70 (15.1)	91 (16.8)
CKD	151 (15.0)	71 (15.3)	80 (14.7)
Liver failure	3 (0.3)	0	3 (0.6)
Symptomatic peripheral vascular disease	43 (4.3)	18 (3.9)	25 (4.6)
Biological heart valve	35 (3.5)	10 (2.2)	25 (4.6)
Atrial fibrillation			
Paroxysmal	237 (23.6)	88 (19.0)	149 (27.4)
Persistent	141 (14.0)	87 (18.8)	54 (9.9)
Long-standing persistent	52 (5.2)	26 (5.6)	26 (4.8)
Permanent	458 (45.5)	216 (46.7)	242 (44.6)
Unknown	118 (11.7)	46 (9.9)	72 (13.3)
**Baseline oral anticoagulation**			
Type of DOAC at the time of index stroke			
Apixaban	369 (36.7)	202 (43.6)	167 (30.8)
Rivaroxaban	346 (34.4)	127 (27.4)	219 (40.3)
Edoxaban	199 (19.8)	92 (19.9)	107 (19.7)
Dabigatran	92 (9.1)	42 (9.1)	50 (9.2)
Time from last DOAC intake to admission, h			
<12	528 (52.5)	250 (54.0)	278 (51.2)
12-24	348 (34.6)	148 (32.0)	200 (36.8)
24-48	130 (12.9)	65 (14.0)	65 (12.0)
DOAC levels on admission available	223 (22.2)	74 (16.0)	149 (27.4)
DOAC levels on admission^b^			
Below range	51 (22.9)	13 (17.6)	38 (25.5)
Within range	160 (71.7)	56 (75.7)	104 (69.8)
Above range	12 (5.4)	5 (6.8)	7 (4.7)
**Medications on admission**			
Antihypertensives	813 (80.8)	372 (80.3)	441 (81.2)
Rhythm control medications	654 (65.0)	291 (62.9)	363 (66.9)
Drug to lower lipid levels	450 (44.7)	213 (46.0)	237 (43.6)
Antidiabetic drug	258 (25.6)	120 (25.9)	138 (25.4)
Antiplatelet drug	83 (8.3)	37 (8.0)	46 (8.5)
Prestroke mRS score, median (IQR)^c^	0 (0.1)	0 (0.1)	0 (0.1)
**Index event characteristics**			
Hospitalization	972 (96.6)	450 (97.2)	522 (96.1)
Hospital setting			
Stroke unit	918 (91.3)	429 (92.7)	489 (90.1)
Intensive care unit	22 (2.2)	11 (2.4)	11 (2.0)
Other hospital unit	66 (6.6)	23 (5.0)	43 (7.9)
NIHSS score on admission, (median, IQR)^d^	9.0 (4.0-17.0)	9.0 (4.0-17.0)	9.0 (4.0-17.0)
Bamford classification			
Total anterior circulation stroke	234 (23.3)	92 (19.9)	142 (26.2)
Partial anterior circulation stroke	591 (58.7)	276 (59.6)	315 (58.0)
Posterior circulation syndrome	96 (9.5)	54 (11.7)	42 (7.7)
Lacunar stroke	85 (8.4)	41 (8.9)	44 (8.1)
Presence of any other TOAST competing etiology other than cardioembolism	254 (25.2)	127 (27.4)	127 (23.4)
Type of competing stroke etiology^e^			
Lacunar	51 (20.1)	30 (23.6)	21 (16.5)
Large artery atherosclerosis	152 (59.8)	72 (56.7)	80 (63.0)
Other determined etiology	32 (12.6)	16 (12.6)	16 (12.6)
Combined competing etiology	19 (7.5)	9 (7.1)	10 (7.9)
Intravenous thrombolysis	93 (9.2)	27 (5.8)	66 (12.2)
Onset to needle time, median (IQR), min	150.0 (95.5-210.0)	153.5 (112.5-197.5)	140.0 (95.0-210.0)
Endovascular treatment	417 (41.5)	187 (40.4)	230 (42.4)
Onset to groin time, median (IQR), min	219.5 (150.0-300.0)	237.0 (160.8-313.8)	207.5 (148.0-276.3)
Intravenous thrombolysis and endovascular treatment	32 (3.2)	7 (1.5)	25 (4.6)
Any hemorrhagic transformation at 24 h^f^	124 (12.3)	49 (10.6)	75 (13.8)
Symptomatic hemorrhagic transformation at 24 h^g^	9 (0.9)	2 (0.4)	7 (1.3)
Time to restart OAC after the index event, median (IQR), d	5 (2-11)	5 (2-10)	6 (3-11)
Anticoagulation discontinued during follow-up	108 (10.7)	49 (10.6)	59 (10.9)
Follow-up time receiving anticoagulation, median (IQR), %	94.0 (88.0-98.0)	94.0 (89.0-98.0)	93.0 (88.0-97.0)
Poststroke medications			
Antihypertensives	827 (82.2)	376 (81.2)	451 (83.1)
Drugs to lower lipid levels	727 (72.3)	326 (70.4)	401 (73.8)
Antidiabetic drugs	256 (25.4)	110 (23.8)	146 (26.9)
Rhythm control drugs	639 (63.5)	285 (61.6)	354 (65.2)
Antiplatelet therapy	139 (13.8)	71 (15.3)	68 (12.5)

Abbreviations: CKD, chronic kidney disease; DOAC, direct oral anticoagulant (OAC); ICH, intracerebral hemorrhage; mRS, modified Rankin Scale; NIHSS, National Institutes of Health Stroke Scale; TIA, transient ischemic attack; TOAST, Trial of Org 10172 in Acute Stroke Treatment; VKA, vitamin K antagonist.

^a^ Includes participants who self-identified as multiracial or as a race not otherwise specified in the registry case report form.

^b^ Includes participants with levels available on admission.

^c^ Scores range from 0 to 6, with higher scores indicating more severe symptoms.

^d^ Scores range from 0 to 42, with higher scores indicating greater stroke severity.

^e^ Includes those with competing etiology other than cardioembolism.

^f^ Defined as any category within the Heidelberg bleeding classification system.

^g^ Defined as any ICH associated with neurologic worsening of at least 4 points on the NIHSS score that is not attributable to other causes.

### Study Outcomes

**Figure 2.**
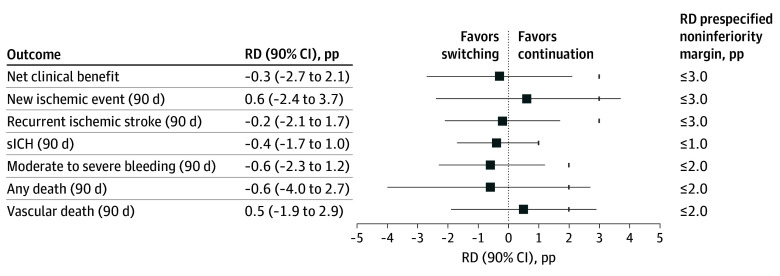
Inverse Propensity Treatment Weighting (IPTW)–Adjusted Noninferiority Analysis of 90-Day Outcomes Squares represent IPTW-adjusted risk differences (RDs), calculated as the weighted event proportion in the group switching direct oral anticoagulant therapy (DOAC) minus that in the group with DOAC therapy continuation. Horizontal bars indicate 90% CIs. Short vertical ticks indicate the prespecified noninferiority margins for each outcome; noninferiority was concluded when the upper bound of the 90% CI did not exceed the corresponding margin. pp Indicates percentage points; sICH, symptomatic intracerebral hemorrhage.

All study outcomes were evaluated in the IPTW population (Figure 2 and eTable 2 in Supplement 1). The 90-day net clinical benefit was similar between switching (4.9%) and continuation groups (5.1%), with an RD of −0.3 pp (90% CI, −2.7 to 2.1 pp), meeting the prespecified noninferiority margin of 3.0%. For new ischemic events 90 days after the index event, the IPTW-adjusted risk was higher in the switching group (8.0%) compared with the continuation group (7.4%), yielding an RD of 0.6 pp (90% CI, −2.4 to 3.7 pp) (Figure 2 and eTable 2 in Supplement 1), precluding formal demonstration of noninferiority. Risk of recurrent ischemic stroke rates at 90 days was also comparable (2.9% vs 3.1%; RD, −0.2 pp [90% CI, −2.1 to 1.7 pp]), supporting noninferiority.

Regarding safety outcomes, the risk of 90-day symptomatic ICH was low in both groups and without any significant difference (0.9% in the switching group vs 1.2% in the continuation group), corresponding to an RD of −0.4 pp (90% CI, −1.7 to 1.0 pp) (Figure 2 and eTable 2 in Supplement 1). The upper bound of the 90% CI remained below the prespecified noninferiority margin of 1.0%, indicating noninferiority on the RD scale. For the risk of 90-day moderate to severe extracranial bleeding, event rates were similar (2.2% vs 2.7%) in the switching vs continuation groups, yielding an RD of −0.6 pp (90% CI, −2.3 pp to 1.2 pp). The 90% CI fell within the prespecified noninferiority margin (2.0%), supporting noninferiority of the continuation strategy (Figure 2 and (eTable 2 in Supplement 1). In contrast, noninferiority was not formally established for the risk of all-cause mortality (8.7% vs 9.3%; RD, −0.6 pp [90% CI, −4.0 to 2.7 pp]) or vascular death (4.9% vs 4.4%; RD, 0.5 pp [90% CI, −1.9 to 2.9 pp]), as 90% CIs exceeded the prespecified margins.

### Association of Poststroke Anticoagulation Strategy With 90-Day Clinical Outcomes

**Figure 3.**
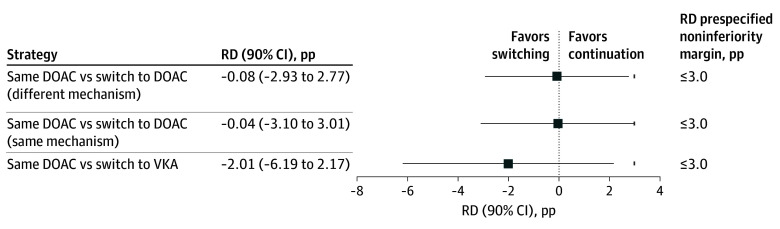
Inverse Probability of Treatment Weighting (IPTW)–Adjusted Noninferiority Comparisons Between Anticoagulation Strategies for 90-Day Net Clinical Benefit Squares represent IPTW-adjusted risk differences (RDs), calculated as the weighted event proportion in each direct oral anticoagulant therapy (DOAC) switching strategy minus that in the DOAC continuation group. Horizontal bars indicate 90% CIs. Short vertical ticks indicate the prespecified noninferiority margin; noninferiority was concluded when the upper bound of the 90% CI did not exceed the predefined margin. pp Indicates percentage points; VKA, vitamin K antagonist.

**Figure 4.**
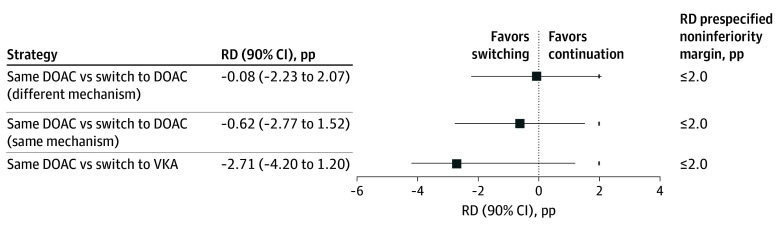
Inverse Probability of Treatment Weighting (IPTW)–Adjusted Noninferiority Comparisons Between Anticoagulation Strategies for 90-Day Moderate to Severe Bleeding Squares represent IPTW-adjusted risk differences (RDs), calculated as the weighted event proportion in each direct oral anticoagulant therapy (DOAC) switching strategy minus that in the DOAC continuation group. Horizontal bars indicate 90% CIs. Short vertical ticks indicate the prespecified noninferiority margin; noninferiority was concluded when the upper bound of the 90% CI did not exceed the predefined margin. pp Indicates percentage points.

When switching to a DOAC with a different mechanism was compared with the continuation strategy (Figure 3 and eTable 3 in Supplement 1), the IPTW-weighted risks were identical (5.1% in both groups), yielding an RD of −0.08 pp (90% CI, −2.93 to 2.77 pp), which met the prespecified noninferiority margin of 3.0% on the absolute scale. However, the corresponding RR (1.02 [90% CI, 0.56-1.72]) exceeded the predefined RR margin (1.59), and noninferiority was therefore not confirmed on the relative scale. When switching to a DOAC with the same mechanism was compared with the continuation strategy, the weighted risks were again similar (5.1% in both groups), resulting in an RD of −0.04 pp (90% CI, −3.10 to 3.01 pp), and noninferiority was not demonstrated. Switching from a DOAC to a VKA was associated with a lower weighted risk in the switching group (3.1% vs 5.1%), corresponding to an RD of −2.01 pp (90% CI, −6.19 to 2.17 pp), which satisfied the noninferiority margin on the RD scale. Figure 4 and eTable 4 in Supplement 1 present the corresponding noninferiority analyses for moderate to severe bleeding at 90 days. Findings of these noninferiority analyses and sensitivity analyses are discussed in the eResults in Supplement 1.

## Discussion

In this emulated target trial of patients with AF who experienced an ischemic stroke while receiving DOAC therapy, switching the anticoagulation agent did not exceed the prespecified noninferiority margins compared with continuation of the same DOAC therapy for most clinically relevant 90-day outcomes. The 90-day net clinical benefit was comparable between groups, with absolute RDs well within the prespecified noninferiority margin. Recurrent ischemic events and bleeding complications were infrequent and similarly distributed across strategies. However, noninferiority was not formally demonstrated for all-cause and vascular mortality, although absolute differences were small and 90% CIs were wide, suggesting limited statistical precision rather than a clear excess risk. Overall, these findings suggest that in patients with breakthrough ischemic stroke while receiving DOACs, switching the DOAC may not be associated with clinically meaningful benefits in short-term outcomes. Our results challenge the empirical practice of switching anticoagulants after a breakthrough stroke.^6,7^ When poor adherence, inappropriate dosing, and alternative stroke mechanisms have been ruled out, continuation of the same DOAC may represent a safer and simpler strategy, avoiding the transient loss of anticoagulant protection, dosing errors, and uncertainty associated with switching.^1,2,4^ Decisions to switch anticoagulation may be justified by patient preference, drug interactions, or specific contraindications but do not represent an established strategy to mitigate the risk of further recurrences. The noninferiority framework allowed us to address the clinically relevant question of whether switching anticoagulation confers a meaningful advantage over continuation, rather than merely testing for statistical differences between groups. In this context, the key concern is not whether the 2 strategies differ, but whether continuation results in an excess risk beyond a clinically acceptable threshold. By prespecifying margins of tolerable risk, this approach provides a direct and interpretable assessment of clinical equivalence in practice. In our analysis, absolute RDs were small and, for most outcomes, remained within the predefined clinically acceptable margins.

Importantly, our findings also have implications for the design and powering of future prospective studies. By explicitly defining noninferiority margins and quantifying event rates for both ischemic and bleeding outcomes, this study provides empirical data that can inform realistic sample size calculations and end point selection for randomized clinical trials evaluating DOAC management after breakthrough stroke. Such trials should be adequately powered to detect modest but clinically meaningful effects and should prioritize the identification of patient subgroups in whom treatment switching may be advantageous, as the present results suggest that most patients are unlikely to derive benefit from a change in therapy.

Our findings are consistent with 2 recent meta-analysis of observational studies.^20,21^ However, by applying a target trial emulation framework with prespecified noninferiority margins, we formally assessed clinical equivalence rather than mere statistical differences. Despite evidence of comparable outcomes, clinical practice remains variable, and many clinicians still opt to switch anticoagulants after a breakthrough stroke.^7^ In our multinational cohort, this pattern was clearly observable: 56.0% of patients were switched to a different anticoagulant regimen after breakthrough stroke, whereas 44.0% continued therapy with the same agent.

An additional finding of this emulated target trial concerns the type of switching strategy. When patients switched to another DOAC, outcomes were similar regardless of whether the alternative agent shared the same mechanism of action or had a different pharmacologic target. In other words, no clinically meaningful differences in 90-day net clinical benefit emerged between switching within the same class and switching across mechanisms. These findings suggest that the act of switching itself—rather than the specific pharmacologic profile of the new DOAC—does not appear to confer a short-term advantage over continuation of the index therapy.

This pattern was consistent across all strategy-specific comparisons. After IPTW, estimated risks were broadly comparable between continuation and each switching approach. Although noninferiority criteria were met in some comparisons and narrowly not met in others, the magnitude of the RDs and direction of the associations did not support a clinically meaningful difference between strategies. The comparison with switching to VKA yielded wider 90% CIs, reflecting the smaller number of patients undergoing this strategy and the low event counts, resulting in reduced precision of the risk estimates. Therefore, while the point estimates remained broadly aligned with the overall pattern, definitive conclusions for this subgroup should be drawn with caution. Taken together, these strategy-specific analyses reinforce the overall finding that no alternative anticoagulation strategy demonstrated clear clinical advantage over continuation of the same DOAC following a breakthrough ischemic stroke. Thus mechanistic switching—for example, from a factor Xa inhibitor to a direct thrombin inhibitor—is unlikely to mitigate the residual cardioembolic risk underlying breakthrough strokes.

The sensitivity analysis excluding patients with a competing stroke etiology further strengthened the robustness of our findings. After removal of these patients, the estimated absolute RD for the 90-day net clinical benefit remained small and within the prespecified noninferiority margin, confirming noninferiority. Notably, the direction of the associations and magnitude of the differences were consistent with the primary analysis in the full cohort, suggesting that the findings were robust to the exclusion of patients with competing causes of stroke. This is particularly relevant because, unlike our approach, most previous observational studies on breakthrough stroke during DOAC therapy did not systematically exclude or account for competing causes.^22,23,24,25,26^ By explicitly addressing this subgroup, our analysis provides additional reassurance that the observed equivalence between the continuation and switching strategies was unlikely to be driven by the presence of a competing stroke etiology, but instead may reflect a true lack of clinically meaningful difference between the 2 approaches in most of the patients.

### Strengths and Limitations

The main strength of this study lies in the use of rigorous procedures to enhance data accuracy and quality, supported by regular quality checks of the ASPERA-R electronic database. The target-trial emulation framework allowed us to address a clinically relevant question not yet tested in randomized clinical trials. However, several limitations merit consideration. Given the noninferiority design, deviations from the initial treatment strategy during follow-up could bias estimates toward noninferiority by diluting between-group differences. Because treatment changes during the 90-day follow-up were not systematically captured across all sites, we could not quantify crossover rates, and residual bias cannot be excluded. Participation was restricted to centers with standardized etiologic testing and structured 90-day follow-up pathways, which enhanced internal validity and consistency of outcome ascertainment but may limit generalizability to settings with less specialized stroke care. The participating centers were predominantly high-volume tertiary stroke units across multiple countries, and our results may therefore be most applicable to similar clinical environments. As such, the study population was representative of patients treated in high-volume, specialized stroke centers, where complex secondary prevention decisions after breakthrough ischemic stroke are most frequently made. In addition, exclusion of patients with missing baseline information or incomplete follow-up may have introduced selection bias, although this approach was necessary to reduce misclassification in a noninferiority framework. Data on concomitant medications potentially interfering with oral anticoagulation were not systematically collected; therefore, they could not be accounted for in the matching procedures at baseline nor formally evaluated for their potential impact on outcomes. Finally, despite emulating a target trial and applying robust statistical methods, causal inference remains constrained by the observational nature of the data and the lack of randomized allocation. Further limitations are reported in the eDiscussion in Supplement 1.

## Conclusions

In this cohort study using an emulated target trial design, continuation of the same DOAC therapy was associated with similar 90-day outcomes compared with switching therapy. Absolute differences were small and generally within prespecified margins, suggesting no clear short-term advantage of routine switching. Randomized studies are needed to define subgroups who may benefit from treatment modification.

## Notes

Supplement 1. **eMethods.** ASPERA Project **eResults.** Primary and Sensitivity Analyses **eDiscussion.** Additional Limitations **eFigure 1.** Study Flow Chart **eFigure 2.** Love Plot **eFigure 3.** Weight Distribution Post Inverse Probability of Treatment Weighting (IPTW) Across the DOAC SAME and DOAC SWITCH Groups **eTable 1.** Covariate Balance Before and After Inverse Probability Weighting (IPW) Between the DOAC SWITCH and DOAC SAME Groups **eTable 2.** Inverse Probability of Treatment Weighting (IPTW)–Adjusted Noninferiority Analysis of 90-Day Outcomes Comparing DOAC SAME and DOAC SWITCH Groups **eTable 3.** Emulated Noninferiority Comparisons Between Anticoagulation Strategies for Net Clinical Benefit Outcome at 90 Days **eTable 4.** Emulated Noninferiority Comparisons Between Anticoagulation Strategies for Outcome of Moderate to Severe Bleeding at 90 Days **eTable 5.** IPTW-Based Sensitivity Analyses for Noninferiority on 90-Day Net Clinical Benefit **eAppendix 1.** Distribution of Patients by Country in the ASPERA-R Study **eAppendix 2.** List of Baseline Variables Collected in the ASPERA-R Study **eAppendix 3.** Protocol Synopsis

Supplement 2.
Data Sharing Statement

## References

[1] Seiffge DJ, Cancelloni V, Räber L, . Secondary stroke prevention in people with atrial fibrillation: treatments and trials. Lancet Neurol. 2024;23(4):404-417. doi:10.1016/S1474-4422(24)00037-1 38508836

[2] Sposato LA, Cameron AC, Johansen MC, . Ischemic stroke prevention in patients with atrial fibrillation and a recent ischemic stroke, TIA, or intracranial hemorrhage: a World Stroke Organization (WSO) scientific statement. Int J Stroke. 2025;20(4):385-400. doi:10.1177/17474930241312649 39719823 PMC11951358

[3] Sposato LA, Sur NB, Katan M, . Embolic stroke of undetermined source: new data and new controversies on cardiac monitoring and anticoagulation. Neurology. 2024;103(1):e209535. doi:10.1212/WNL.0000000000209535 38861698

[4] D’Anna L, Filippidis FT, Harvey K, Korompoki E, Veltkamp R. Ischemic stroke in oral anticoagulated patients with atrial fibrillation. Acta Neurol Scand. 2022;145(3):288-296. doi:10.1111/ane.13552 34766621

[5] Lin SY, Liao YT, Tang SC, Lin CCC, Wang CC. Changing or retaining direct oral anticoagulant after ischemic stroke despite direct oral anticoagulant treatment. J Am Heart Assoc. 2024;13(3):e032454. doi:10.1161/JAHA.123.032454 38293918 PMC11056173

[6] Salehi Omran S, Parikh NS, Zambrano Espinoza M, . Managing ischemic stroke in patients already on anticoagulation for atrial fibrillation: a nationwide practice survey. J Stroke Cerebrovasc Dis. 2020;29(12):105291. doi:10.1016/j.jstrokecerebrovasdis.2020.105291 32992194

[7] Adelakun AR, Turgeon RD, De Vera MA, McGrail K, Loewen PS. Oral anticoagulant switching in patients with atrial fibrillation: a scoping review. BMJ Open. 2023;13(4):e071907. doi:10.1136/bmjopen-2023-071907 37185198 PMC10151984

[8] Hernán MA, Robins JM. Using big data to emulate a target trial when a randomized trial is not available. Am J Epidemiol. 2016;183(8):758-764. doi:10.1093/aje/kwv254 26994063 PMC4832051

[9] Hernán MA, Sauer BC, Hernández-Díaz S, Platt R, Shrier I. Specifying a target trial prevents immortal time bias and other self-inflicted injuries in observational analyses. J Clin Epidemiol. 2016;79:70-75. doi:10.1016/j.jclinepi.2016.04.014 27237061 PMC5124536

[10] Advancing knowledge in ischemic stroke patients on oral anticoagulants (ASPERA). ClinicalTrials.gov identifier: NCT06823466. Updated February 20, 2025. Accessed March 24, 2026. https://clinicaltrials.gov/study/NCT06823466

[11] Foschi M, De Santis F, Gabriele F, . mpact of cancer on outcomes following breakthrough ischaemic stroke on oral anticoagulants for atrial fibrillation: insights from the ASPERA-R study. Eur Stroke J. Published online February 9, 2026. doi:10.1093/esj/aakag015 PMC12947708 41758563

[12] GUSTO investigators. An international randomized trial comparing four thrombolytic strategies for acute myocardial infarction. N Engl J Med. 1993;329(10):673-682. doi:10.1056/NEJM199309023291001 8204123

[13] Hacke W, Kaste M, Fieschi C, ; Second European-Australasian Acute Stroke Study Investigators. Randomised double-blind placebo-controlled trial of thrombolytic therapy with intravenous alteplase in acute ischaemic stroke (ECASS II). Lancet. 1998;352(9136):1245-1251. doi:10.1016/S0140-6736(98)08020-9 9788453

[14] Stuart EA. Matching methods for causal inference: a review and a look forward. Stat Sci. 2010;25(1):1-21. doi:10.1214/09-STS313 20871802 PMC2943670

[15] The European Agency for the Evaluation of Medicinal Products evaluation of medicines for human use. European Medicines Agency. 2000. http://www.eudra.org/emea.html

[16] Choice of control group and related issues in clinical trials E10. International Conference on Harmonisation of Technical Requirements for Registration of Pharmaceuticals for Human Use. July 20, 2000. Accessed March 24, 2026. https://database.ich.org/sites/default/files/E10_Guideline.pdf

[17] Hankey GJ. Secondary stroke prevention. Lancet Neurol. 2014;13(2):178-194. doi:10.1016/S1474-4422(13)70255-2 24361114

[18] Guideline on the choice of the non-inferiority margin. European Medicines Agency. July 27, 2005. Accessed March 24, 2026. https://www.ema.europa.eu/en/documents/scientific-guideline/guideline-choice-non-inferiority-margin_en.pdf

[19] ICH E9(R1) addendum on estimands and sensitivity analysis in clinical trials. European Medicines Agency. February 17, 2020. Accessed March 24, 2026. https://www.ema.europa.eu/en/documents/scientific-guideline/ich-e9-r1-addendum-estimands-and-sensitivity-analysis-clinical-trials-guideline-statistical-principles-clinical-trials-step-5_en.pdfx

[20] Mota Telles JP, Cenci GI, Marinheiro G, . Anticoagulation strategy for patients presenting with ischemic strokes while using a direct oral anticoagulant: a systematic review and meta-analysis. Int J Stroke. 2025;20(1):42-52. doi:10.1177/17474930241270443 39075753

[21] Romoli M, Paciaroni M, Marrone N, . Anticoagulation strategies following breakthrough ischemic stroke while on direct anticoagulants: a meta-analysis. Neurology. 2025;105(4):e213964. doi:10.1212/WNL.0000000000213964 40758940

[22] Hsieh MT, Liu CH, Lin SH, . Comparing efficacy and safety between patients with atrial fibrillation taking direct oral anticoagulants or warfarin after direct oral anticoagulant failure. J Am Heart Assoc. 2023;12(23):e029979. doi:10.1161/JAHA.123.029979 38038171 PMC10727336

[23] Polymeris AA, Meinel TR, Oehler H, . Aetiology, secondary prevention strategies and outcomes of ischaemic stroke despite oral anticoagulant therapy in patients with atrial fibrillation. J Neurol Neurosurg Psychiatry. 2022;93(6):588-598. doi:10.1136/jnnp-2021-328391 35396339 PMC9148984

[24] Duong E, Lin M, Hodgson M, Jickling G, George-Phillips K, Bungard TJ. Choice of oral anticoagulant: outcomes in atrial fibrillation patients post-stroke despite direct oral anticoagulant use. CJC Open. 2023;5(8):603-610. doi:10.1016/j.cjco.2023.05.001 37720181 PMC10502439

[25] Grifoni E, Pagni B, Sansone T, . Clinical features, management, and recurrence of acute ischemic stroke occurring in patients on oral anticoagulant treatment for nonvalvular atrial fibrillation: a real-world retrospective study. Neurologist. 2024;29(6):329-338. doi:10.1097/NRL.0000000000000579 39344366

[26] Ip YMB, Lau KK, Ko H, . Association of alternative anticoagulation strategies and outcomes in patients with ischemic stroke while taking a direct oral anticoagulant. Neurology. 2023;101(4):e358-e369. doi:10.1212/WNL.0000000000207422 37225430 PMC10435051

