# Supplementary material for: Continuation vs Switching Direct Oral Anticoagulant Therapy After Breakthrough Stroke
Source: JAMA Netw Open. 2026 Apr 28;9(4):e269584. doi: 10.1001/jamanetworkopen.2026.9584 (PMC13126222; doi:10.1001/jamanetworkopen.2026.9584)
Supplement: Supplement 1. — eMethods. ASPERA Project eResults. Primary and Sensitivity Analyses eDiscussion. Additional Limitations eFigure 1. Study Flow Chart eFigure 2. Love Plot eFigure 3. Weight Distribution Post Inverse Probability of Treatment Weighting (IPTW) Across the DOAC SAME and DOAC SWITCH Groups eTable 1. Covariate Balance Before and After Inverse Probability Weighting (IPW) Between the DOAC SWITCH and DOAC SAME Groups eTable 2. Inverse Probability of Treatment Weighting (IPTW)–Adjusted Noninferiority Analysis of 90-Day Outcomes Comparing DOAC SAME and DOAC SWITCH Groups eTable 3. Emulated Noninferiority Comparisons Between Anticoagulation Strategies for Net Clinical Benefit Outcome at 90 Days eTable 4. Emulated Noninferiority Comparisons Between Anticoagulation Strategies for Outcome of Moderate to Severe Bleeding at 90 Days eTable 5. IPTW-Based Sensitivity Analyses for Noninferiority on 90-Day Net Clinical Benefit eAppendix 1. Distribution of Patients by Country in the ASPERA-R Study eAppendix 2. List of Baseline Variables Collected in the ASPERA-R Study eAppendix 3. Protocol Synopsis [file jamanetwopen-e269584-s001.pdf]

Supplementary Online Content

D’Anna L, Gabriele F, Ornello R, et al. Continuation vs switching direct oral anticoagulant therapy after breakthrough stroke. *JAMA Netw Open.* 2026;9(4):e269584. doi:10.1001/jamanetworkopen.2026.9584

eMethods. ASPERA Project
eResults. Primary and Sensitivity Analyses
eDiscussion. Additional Limitations
eFigure 1. Study Flow Chart
eFigure 2. Love Plot
eFigure 3. Weight Distribution Post Inverse Probability of Treatment Weighting (IPTW) Across the DOAC SAME and DOAC SWITCH Groups
eTable 1. Covariate Balance Before and After Inverse Probability Weighting (IPW) Between the DOAC SWITCH and DOAC SAME Groups
eTable 2. Inverse Probability of Treatment Weighting (IPTW)–Adjusted Noninferiority Analysis of 90-Day Outcomes Comparing DOAC SAME and DOAC SWITCH Groups
eTable 3. Emulated Noninferiority Comparisons Between Anticoagulation Strategies for Net Clinical Benefit Outcome at 90 Days
eTable 4. Emulated Noninferiority Comparisons Between Anticoagulation Strategies for Outcome of Moderate to Severe Bleeding at 90 Days
eTable 5. IPTW-Based Sensitivity Analyses for Noninferiority on 90-Day Net Clinical Benefit
eAppendix 1. Distribution of Patients by Country in the ASPERA-R Study
eAppendix 2. List of Baseline Variables Collected in the ASPERA-R Study
eAppendix 3. Protocol Synopsis

This supplementary material has been provided by the authors to give readers additional information about their work.

© 2026 D’Anna L et al. *JAMA Network Open*.

eMethods. ASPERA Project

Eligible patients were required to have a breakthrough ischemic event—either first-ever or recurrent—diagnosed according to World Health Organization (WHO) criteria and confirmed by non-contrast computed tomography (NCCT) and/or magnetic resonance imaging (MRI). The index stroke had to occur during continuous treatment with an oral anticoagulant, either a direct oral anticoagulant (DOAC) or a vitamin K antagonist (VKA). In addition, eligibility required the availability of baseline extracranial and intracranial vascular imaging, including computed tomography angiography (CTA), magnetic resonance angiography (MRA), color Doppler ultrasonography (CDU), or digital subtraction angiography (DSA), as well as a 90 ± 10-day follow-up after the index event with standardized outcome ascertainment. Patients were excluded if anticoagulation therapy was considered inadequate, defined as DOAC regimens not meeting guideline-recommended criteria based on renal function, age, body weight, or relevant drug interactions, including underdosing or unjustified dose reductions. Patients were also excluded in the presence of poor compliance with anticoagulant therapy, defined as missed or uncertain intake of oral anticoagulants during the 7 days preceding the stroke, as documented in medical records or reported by the patient or caregiver. All consecutive admissions to hospital wards or evaluations in emergency departments during the study period were screened to minimize selection bias. Data were systematically extracted from hospital records and entered into the registry using a standardized Research Electronic Data Capture (REDCap) case report form (CRF) to ensure consistency and accuracy. The CRF included predefined mandatory variables required for eligibility, exposure classification, outcome ascertainment, and primary analyses, as well as optional variables collected for exploratory purposes. The full list of both mandatory and optional baseline variables is provided in Appendix S2. Missing or inconsistent mandatory data triggered prespecified central queries before database lock. To maintain data quality, the ASPERA-R electronic database was subject to weekly checks and central monitoring procedures. Aggregated data can be provided upon reasonable request.

© 2026 D’Anna L et al. *JAMA Network Open*.

eResults. Primary and Sensitivity Analyses

Table S4 and Figure 4 present the corresponding non-inferiority analyses for moderate–severe bleeding at 90 days (see supplemental results). When switching to a DOAC with a different mechanism was compared with $DOAC_{SAME}$, the weighted risks were similar (2.6% vs 2.7%), resulting in a risk difference of −0.08% (90% CI −2.23 to 2.07). The upper bound exceeded the prespecified RD margin of 2.0%, and non-inferiority was therefore not demonstrated. The risk ratio (0.98, 90% CI 0.41 to 2.34) also failed to satisfy the RR-based criterion. In contrast, switching to a DOAC with the same mechanism yielded a weighted risk difference of −0.62% (90% CI −2.77 to 1.52) compared with $DOAC_{SAME}$, with the upper confidence bound remaining within the 2.0% margin, thus meeting the non-inferiority criterion on the RD scale. The corresponding risk ratio (0.78, 90% CI 0.31 to 1.95) also met the predefined RR threshold. Switching to a vitamin K antagonist was associated with a lower weighted bleeding risk in the switch group (0.0% vs 2.7%), resulting in a risk difference of −2.71% (90% CI −4.20 to 1.20), which satisfied the RD non-inferiority margin. However, due to the absence of events in the VKA group and consequent instability of the relative estimate, non-inferiority could not be formally established on the RR scale.

Sensitivity analyses

Table S5 shows the results of multiple sensitivity analyses evaluating the robustness of the non-inferiority findings for the 90-day net clinical benefit outcome. Across all weighting approaches, the risk differences (defined as pSWITCH − pSAME) remained negative and well within the prespecified non-inferiority margin of 3.0%, indicating that switching anticoagulation was not associated with a clinically unacceptable increase in risk compared with continuation of the same DOAC. The primary ATE estimate yielded a risk difference of −0.26% (90% CI −2.65 to 2.14), confirming that any potential difference in net clinical benefit did not exceed the acceptable loss threshold. Similar results were observed when applying trimming at 1–99% and 5–95% of the propensity score distribution, overlap weighting (ATO), and covariate-adjusted regression without weighting, as well as in the per-protocol sensitivity analysis restricted to patients who restarted anticoagulation within 10 days, and in the augmented IPTW estimator. In contrast, none of the analyses met the non-inferiority criterion on the relative scale. Although all estimated risk ratios were close to unity, the upper bounds of the 90% confidence intervals exceeded the pre-specified relative margin ($RR \leq 1.30$), and therefore non-inferiority could not be demonstrated when using the RR framework. In a sensitivity analysis excluding patients with a competing stroke etiology, the IPTW-adjusted

© 2026 D'Anna L et al. *JAMA Network Open*.

risk difference for net clinical benefit at 90 days was −0.92% (90% CI −3.59 to 1.76), remaining within the prespecified non-inferiority margin of 3.0%. The corresponding risk ratio was 0.81 (90% CI 0.45–1.47), which did not meet the RR non-inferiority criterion (RR ≤ 1.30).

© 2026 D'Anna L et al. *JAMA Network Open*.

eDiscussion. Additional Limitations

Despite rigorous IPW and additional adjustment for residually imbalanced covariates, the observational design of ASPERA precludes ruling out residual confounding. Second, the retrospective nature may introduce selection and measurement bias and limits causal inference. Third, plasma DOAC concentrations were available only for a small subset of patients with breakthrough ischemic stroke, precluding robust adjustment for on-treatment exposure; therefore, for most patients these parameters were estimated from clinical history and chart review, potentially introducing misclassification and recall/documentation bias. Fourth, patients with unavailable 90-day follow-up data or with incomplete information in at least one mandatory baseline variable were excluded from the analysis. although they represented only a small percentage of the initial cohort and we found no evidence of differential or non-random attrition across countries, selection bias cannot be entirely excluded. Most patients were enrolled in Italy (60.0%); although country of enrollment was included in the matching strategy, this geographic concentration may limit generalizability and leave room for country-level residual confounding. Consequently, the cohort was strongly imbalanced toward the non-Hispanic white ethnicity, which may further restrict generalizability outside this group. Although we excluded patients with overt non-adherence or inappropriate dosing, subtle differences in compliance or dosing intervals might have persisted and contributed to apparent treatment failures. The relatively small number of patients that switched from DOAC to VKAs limited the precision of estimates for these subgroups. Our analysis focused on short-term (90-day) outcomes; the long-term consequences of maintaining versus switching anticoagulation strategies remain uncertain and warrant further evaluation. The clinical decisions surrounding DOAC switching may have been influenced by unrecorded factors—such as patient preference, frailty, renal function, or prescriber experience—that could not be fully accounted for in the models.

eFigure 1. Study Flow Chart

© 2026 D'Anna L et al. *JAMA Network Open*.

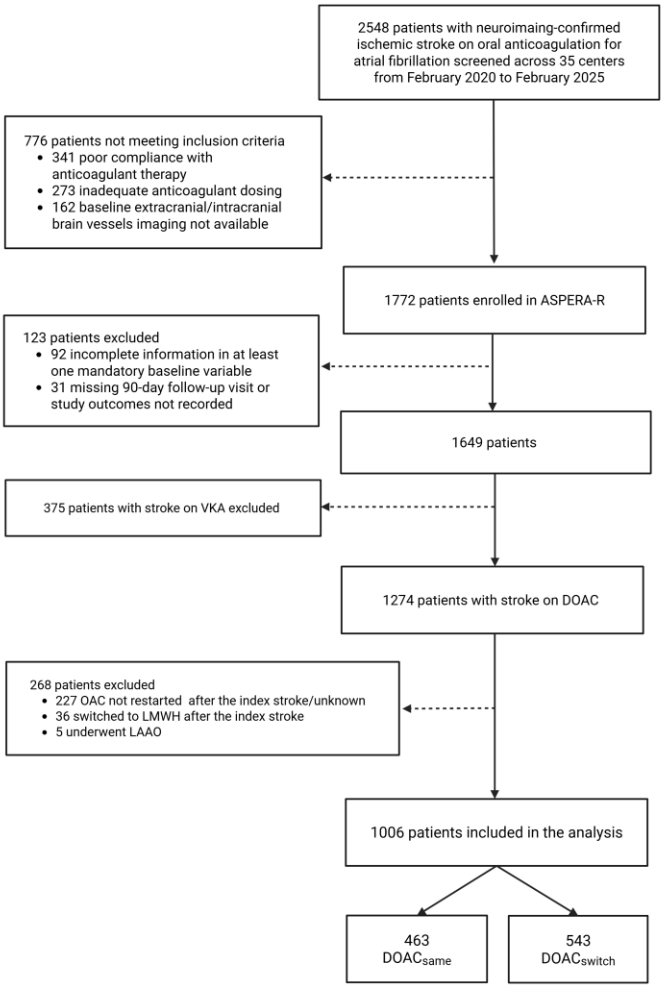

© 2026 D’Anna L et al. *JAMA Network Open*.

eFigure 2. Love Plot

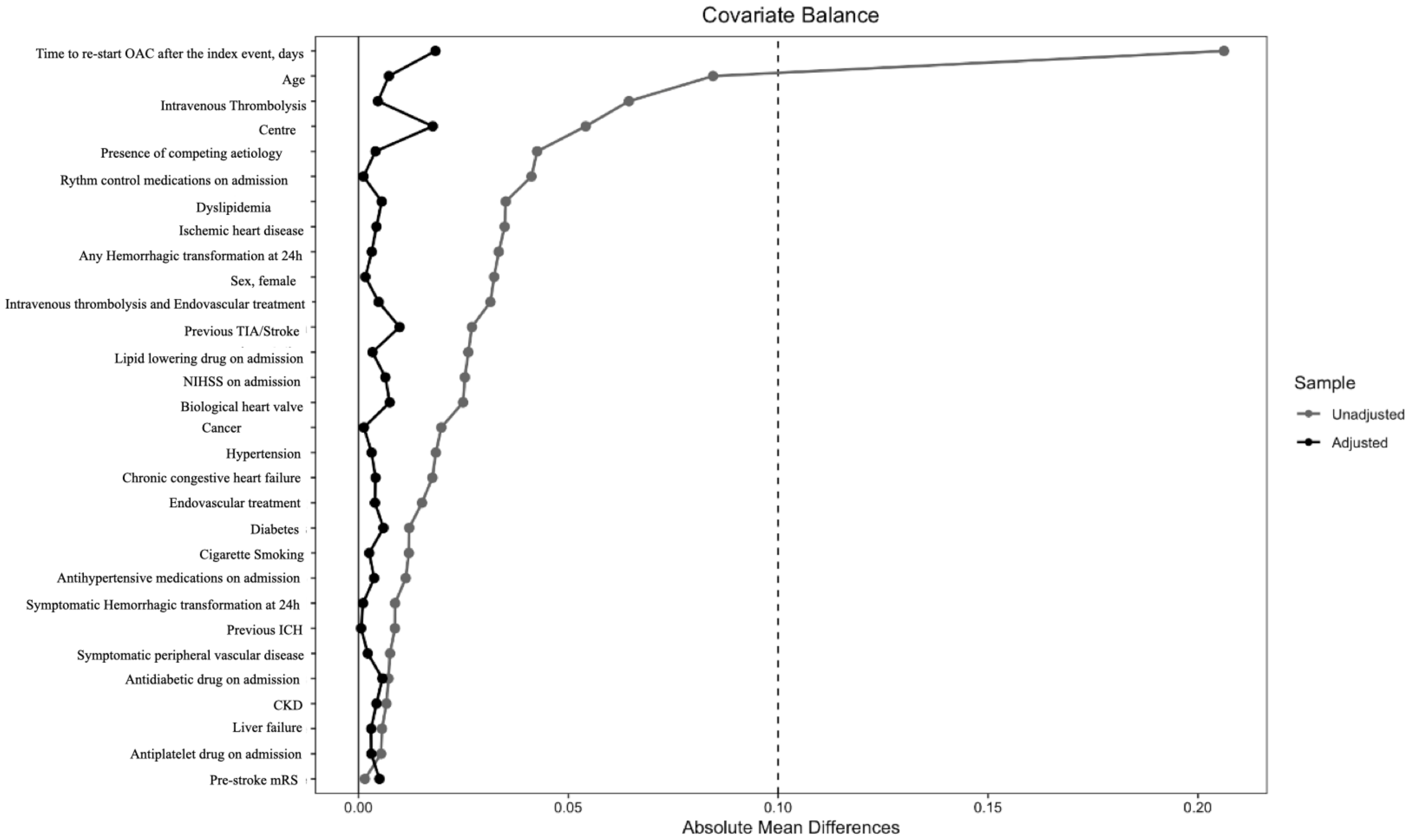

© 2026 D'Anna L et al. *JAMA Network Open*.

eFigure 3. Weight Distribution Post Inverse Probability of Treatment Weighting (IPTW) Across the DOAC SAME and DOAC SWITCH Groups

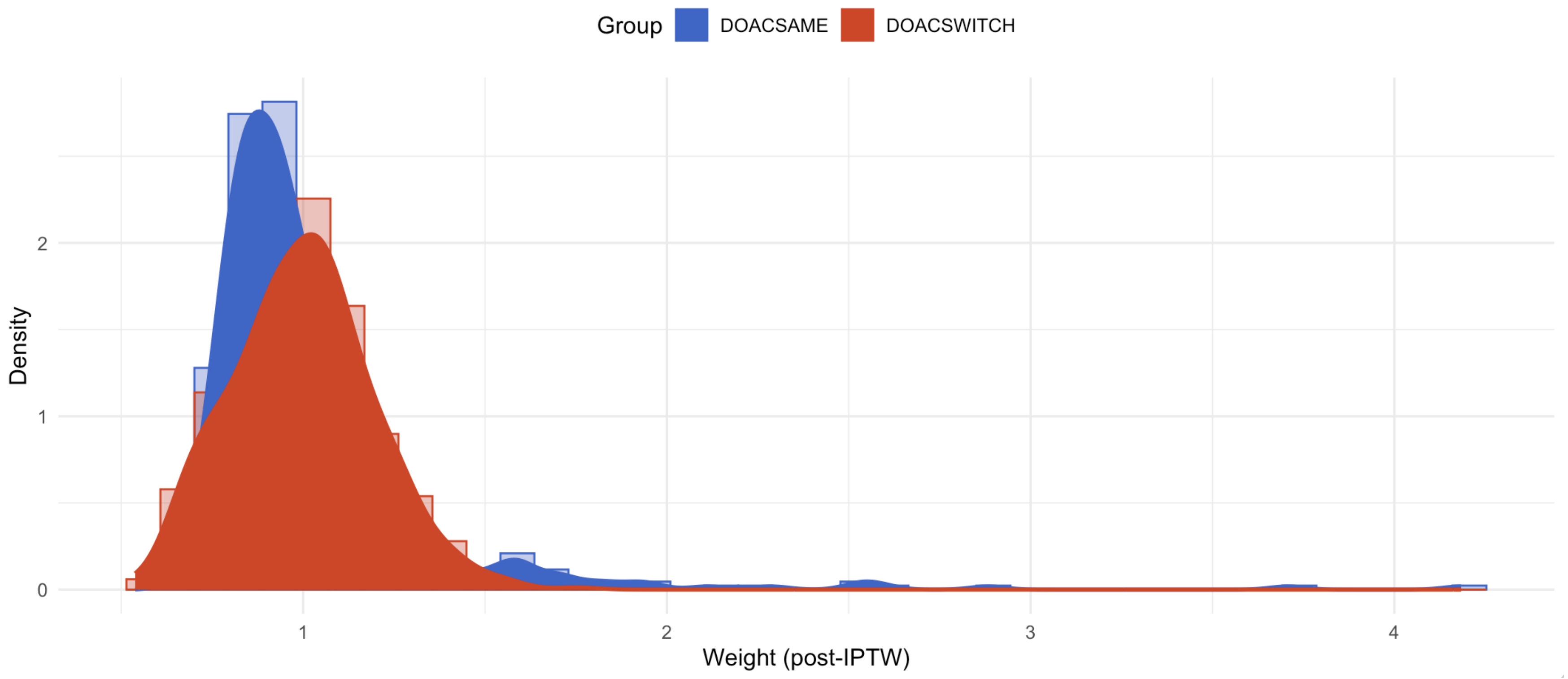

© 2026 D’Anna L et al. *JAMA Network Open*.

eTable 1. Covariate Balance Before and After Inverse Probability Weighting (IPW) Between the DOAC SWITCH and DOAC SAME Groups

	**SMD unweighted**	**SMD weighted**
Center	0.054	0.018
Age, years	0.084	0.007
Sex, female	0.032	0.002
Smoking	0.012	0.003
Hypertension	0.018	0.003
Dyslipidemia	0.035	0.006
Diabetes	0.012	0.006
Prior myocardial infarction	0.035	0.004
Cancer	0.020	0.001
Previous TIA/Stroke	0.027	0.010
Previous ICH	0.009	0.001
Heart failure	0.018	0.004
CKD	0.007	0.004
Liver failure	0.006	0.003
Peripheral vascular disease	0.008	0.002
Biological heart valve	0.025	0.007
Previous use of antihypertensive medications	0.011	0.004
Previous use of antirythm medications	0.041	0.001
Previous use of statin medications	0.026	0.003
Previous use of antidiabetic medications	0.007	0.006
Previous use of antiplatelet medications	0.005	0.003

© 2026 D’Anna L et al. *JAMA Network Open*.

Pre-stroke mRS	0.002	0.005
NIHSS on admission	0.025	0.006
Presence of any other TOAST competing etiology	0.043	0.004
Intravenous thrombolysis	0.064	0.005
Endovascular treatment	0.015	0.004
Intravenous thrombolysis and Endovascular treatment	0.031	0.005
Any Hemorrhagic transformation	0.033	0.003
Symptomatic Hemorrhagic transformation	0.009	0.001
Time to re-start OAC after the index event, days	0.206	0.018

Values are expressed as n (%) for categorical variables and as median (interquartile range, IQR) for continuous variables. Standardized mean differences (SMDs) were calculated to assess covariate balance before (unweighted) and after (weighted) application of IPW. An absolute SMD < 0.10 was considered indicative of adequate balance, and all covariates achieved excellent balance after weighting (all SMDs < 0.01). Abbreviations: OAC, oral anticoagulant; ICH, intracerebral hemorrhage; CKD, chronic kidney disease; TIA, transient ischemic attack; mRS, modified Rankin Scale; NIHSS, National Institutes of Health Stroke Scale; TOAST, Trial of Org 10172 in Acute Stroke Treatment.

© 2026 D'Anna L et al. *JAMA Network Open*.

eTable 2. Inverse Probability of Treatment Weighting (IPTW)–Adjusted Noninferiority Analysis of 90-Day Outcomes Comparing DOAC SAME and DOAC SWITCH Groups

	DOACSAME n/N (%)	DOACSWITCH n/N (%)	DOACSAME Post IPTW (%)	DOACSWITCH Post IPTW (%)	Post IPTW RD (90% CI)	Margin (RD ≤)	NI satisfied	Post IPTW RR (90% CI)	Margin (RR ≤)	NI satisfied
**Primary outcome**										
Net clinical benefit	23/463 (5.0)	29/543 (5.3)	5.1	4.9	-0.3% (-2.7 to 2.1)	3.0%	Yes	0.95 (0.59 to 1.52)	1.61	Yes
**Secondary outcome**										
New ischemic event at 90 days	32/463 (6.9)	46/543 (8.5)	7.4	8.0	0.6% (-2.4 to 3.7)	3.0%	No	1.08 (0.73 to 1.61)	1.37	No
Recurrent ischemic stroke at 90 days	13/463 (2.8)	17/543 (3.1)	3.1	2.9	-0.2% (-2.1 to 1.7)	3.0%	Yes	0.93 (0.49 to 1.77)	2.05	Yes
**Safety outcome**										
sICH at 90 days	4/463 (0.9)	6/543 (1.1)	1.2	0.9	-0.4% (-1.7 to 1.0)	1.0%	Yes	0.71 (0.22 to 2.29)	2.15	No

© 2026 D'Anna L et al. *JAMA Network Open*.

Moderate-severe bleeding at 90 days	11/463 (2.4)	13/543 (2.4)	2.7	2.2	-0.6 (-2.3 to 1.2)	2.0%	Yes	0.80 (0.39 to 1.63)	1.93	Yes
Any death at 90 days	40/463 (8.6)	48/543 (8.8)	9.3	8.7	-0.6 (-4.0 to 2.7)	2.0%	No	0.93 (0.64 to 1.35)	1.23	No
Vascular death at 90 days	19/463 (4.1)	27/543 (5.0)	4.4	4.9	0.5 (-1.9 to 2.9)	2.0%	No	1.11 (0.65 to 1.87)	1.41	No

Values are reported as unweighted counts (n/N, %) and inverse probability of treatment weighting (IPTW)–adjusted event rates. Risk Differences (RD) represent p(DOAC-SWITCH) − p(DOAC-SAME). Non-inferiority was concluded when the upper bound of the 90% confidence interval for the RD was less than or equal to the prespecified non-inferiority margin for that outcome (3.0% for ischemic outcomes, 2.0% for major bleeding, 1.0% for intracranial hemorrhage, and 2.0% for mortality). Risk Ratios (RR) were estimated using IPTW-weighted log-binomial models; non-inferiority was concluded when the upper bound of the 90% CI was ≤ the prespecified RR margin. NI satisfied = overall non-inferiority conclusion for each outcome.

© 2026 D'Anna L et al. *JAMA Network Open*.

eTable 3. Emulated Noninferiority Comparisons Between Anticoagulation Strategies for Net Clinical Benefit Outcome at 90 Days

Comparison	DOACSAME n/N (%)	DOACSWITCH n/N (%)	DOACSAME Post IPTW (%)	DOACSWITCH Post IPTW (%)	Post IPTW RD (90% CI)	Margin (RD ≤)	NI (RD)	Post IPTW RR (90% CI)	Margin (RR ≤)	NI (RR)
Same DOAC vs switch to a DOAC with a different mechanism	23/463 (5.0)	15/283 (5.3)	5.1	5.1	-0.08 (-2.93 to 2.77)	3.0	Yes	1.02 (0.56 to 1.72)	1.59	No
Same DOAC vs switch to a DOAC with same mechanism	23/463 (5.0)	5/201 (2.5)	5.1	5.1	-0.04 (-3.10 to 3.01)	3.0	No	1.01 (0.55 to 1.80)	1.59	No
Same DOAC vs switch to VKA	23/463 (5.0)	0/59 (0.0)	5.1	3.1	-2.01 (-6.19 to 2.17)	3.0	Yes	0.61 (0.18 to 2.12)	1.96	No

RD indicates the risk difference, defined as the weighted proportion of events in the comparison group minus that in the DOACSAME group (RD = pSWITCH − pSAME). RR indicates the risk ratio, calculated as pSWITCH / pSAME. Non-inferiority for RD was concluded if the upper bound of the 90% confidence interval was less than or equal to the pre-specified non-inferiority margin (3.0% for net clinical benefit), indicating that switching did not increase the absolute risk beyond the allowed margin. Non-inferiority for RR was assessed similarly using predefined margins (RR ≤ 1.59 for DOAC-to-DOAC comparisons and RR ≤ 1.96 for DOAC-to-VKA). IPTW = inverse probability of treatment weighting.

© 2026 D'Anna L et al. *JAMA Network Open*.

eTable 4. Emulated Noninferiority Comparisons Between Anticoagulation Strategies for Outcome of Moderate to Severe Bleeding at 90 Days

Comparison	DOACSAME n/N (%)	DOACSWITCH n/N (%)	DOACSAME Post IPTW (%)	DOACSWITCH Post IPTW (%)	Post IPTW RD (90% CI)	Margin (RD ≤)	NI (RD)	Post IPTW RR (90% CI)	Margin (RR ≤)	NI (RR)
Same DOAC vs switch to a DOAC with a different mechanism	11/463 (2.4)	8/283 (5.3)	2.7	2.6	-0.08 (-2.23 to 2.07)	2.0	No	0.98 (0.41 to 2.34)	1.59	No
Same DOAC vs switch to a DOAC with same mechanism	11/463 (2.4)	5/201 (6.0)	2.7	2.1	-0.62 (-2.77 to 1.52)	2.0	Yes	0.78 (0.31 to 1.95)	1.59	Yes
Same DOAC vs switch to VKA	11/463 (2.4)	0/59 (0)	2.7	0.0	-2.71 (-4.20 to 1.20)	2.0	Yes	0.00	1.96	No

RD indicates the risk difference, defined as the IPTW-weighted proportion of events in the comparison group minus that in the DOACSAME group (RD = p SWITCH – p SAME). RR indicates the risk ratio, calculated as p SWITCH / p SAME. For this bleeding outcome, non-inferiority for RD was concluded if the upper bound of the 90% confidence interval was less than or equal to the pre-specified non-inferiority margin of 2.0%, indicating that switching did not increase the absolute risk of moderate–severe bleeding beyond the accepted tolerance. Non-inferiority for RR was evaluated analogously using predefined RR margins, derived from the RD margin (RR ≤ 1.59 for DOAC-to-DOAC comparisons and RR ≤ 1.96 for DOAC-to-VKA comparisons). IPTW = inverse probability of treatment weighting.

© 2026 D'Anna L et al. *JAMA Network Open*.

eTable 5. IPTW-Based Sensitivity Analyses for Noninferiority on 90-Day Net Clinical Benefit

Sensitivity	RD (90% CI)	NI Margin (RD)	NI (RD)	RR (90% CI)	NI Margin (RR)	NI (RR)
ATE IPTW (baseline)	-0.26% (-2.65 to 2.14)	≤ 3.0%)	Yes	0.95 (0.59 to 1.52)	≤ 1.30	No
Trimming 1-99%	-0.56% (-2.99 to 1.87)	≤ 3.0%)	Yes	0.89 (0.55 to 1.44)	≤ 1.30	No
Trimming 5-95%	-0.77% (-3.06 to 1.52)	≤ 3.0%)	Yes	0.84 (0.51 to 1.40)	≤ 1.30	No
Overlap weights (ATO)	-0.48% (-2.73 to 1.76)	≤ 3.0%)	Yes	0.91 (0.57 to 1.44)	≤ 1.30	No
Unweighted covariate-adjusted	-0.75% (-3.40 to 1.91)	≤ 3.0%)	Yes	0.87 (0.53 to 1.36)	≤ 1.30	No
Per-protocol restart OAC ≤ 10 days	-0.35% (-2.28 to 1.58)	≤ 3.0%)	Yes	0.88 (0.43 to 1.81)	≤ 1.30	No
AIPW (bootstrap 200)	-0.54% (-3.24 to 2.16)	≤ 3.0%)	Yes	0.90 (0.54 to 1.46)	≤ 1.30	No
Excluding competing etiology	-0.92% (-3.59 to 1.76)	≤ 3.0%)	Yes	0.81 (0.45 to 1.47)	≤ 1.30	No

Abbreviations: ATE = average treatment effect; IPTW = inverse probability of treatment weighting; ATO = average treatment effect in the overlap population; AIPW = augmented inverse probability weighting; OAC = oral anticoagulation; RD = risk difference; RR = relative risk; NI = non-inferiority.

© 2026 D'Anna L et al. *JAMA Network Open*.

eAppendix 1. Distribution of Patients by Country in the ASPERA-R Study

Country	Enrolling center(s)	No. of patients/total no. included in the ASPERA-R(%)	No. of patients/total no. included in the study analysis (%)
Italy	• Stroke Unit and Neurology Unit, S.S. Filippo e Nicola Hospital, Avezzano.<br>• Stroke Unit, Maggiore Hospital, Bologna.<br>• Neurology Unit, IRCCS Policlinico S.Orsola-Malpighi, Bologna.<br>• Stroke Unit, IRCCS Policlinico Universitario Agostino Gemelli, Rome.<br>• Stroke Unit – Neurology Unit, S.Maria delle Croci Hospital, Ravenna.<br>• Stroke Unit, ASST Grande Ospedale Metropolitano Niguarda, Milano.<br>• Stroke Unit, Luigi Sacco Hospital, Milano.<br>• Stroke Unit, Policlinico San Matteo, Pavia.<br>• Stroke Unit – Neurology Unit, Vito Fazzi Hospital, Lecce.<br>• Stroke Unit, Fabrizio Spaziani Hospital, Frosinone.<br>• Stroke Unit, S.Maria della Misericordia Hospital, Perugia.<br>• Stroke Unit, Azienda Ospedaliera Ospedali Riuniti Villa Sofia – Cervello, Palermo.<br>• Stroke Unit, Arcispedale Santa Maria Nuova, Reggio Emilia.<br>• Stroke Unit, AORN Cardarelli Hospital, Napoli.<br>• Stroke Unit, Azienda Ospedaliero Universitaria Careggi, Firenze.<br>• Stroke Unit, S.Maria della Misericordia Hospital, Udine.<br>• Stroke Unit, Ospedale Riuniti di Ancona, Ancona.<br>• Stroke Unit, Maggiore Hospital, Crema.<br>• Stroke Unit – Neurology Unit, "Di Venere" Hospital, Bari.	1010/1772 (57.0)	989/1649 (60.0)
France	• Stroke Unit, Université Côte d'Azur, Nice.	79/1772 (4.5)	78/1649 (4.7)
Germany	• Stroke Unit – Neurology Unit, Martin Luther University, Halle-Wittenberg.<br>• Stroke Unit, Saarland University Hospital, Homburg.	67/1772 (3.8)	43/1649 (2.6)
Spain	• Stroke Unit, Hospital Universitario La Paz, Madrid	81/1772 (4.6)	80/1649 (4.9)
Portugal	• Stroke Unit, São José Hospital, Lisbon.<br>• Stroke Unit, Hospital de Santa Maria, Lisbon.	124/1772 (7.0)	101/1649 (6.1)
United Kingdom	• Stroke Unit, Charing Cross Hospital, London.<br>• Stroke Unit, St George University Hospital, London.<br>• Stroke Unit, North Bristol NHS Trust, Bristol.	180/1772 (10.2)	178/1649 (10.8)
Croatia	• Stroke Unit, Sveti Duh University Hospital, Zagreb.	35/1772 (19.7)	32/1649 (19.4)
Egypt and Saudi Arabia	• Stroke Unit – Neurology Unit, Aim Shams University Hospital, Cairo.<br>• Stroke Unit, Armed Forces Medical Complex Kobry El Kobba, Cairo.<br>• Stroke Unit, Cairo University Hospital, Cairo.<br>• Stroke Unit, Assiut University Hospital, Asyut.<br>• Stroke Unit – Neurology Unit, King Fahd Hospital, Riyahd.	196/1772 (11.1)	148/1949 (9.0)

© 2026 D'Anna L et al. *JAMA Network Open*.

eAppendix 2. List of Baseline Variables Collected in the ASPERA-R Study

Variable	Mandatory	Notes
**Demographics**		
Date of index ischemic stroke on oral anticoagulation	Yes	-
Hospitalization (Yes/No)	Yes	-
Hospitalization setting<br>- Stroke unit<br>- Intensive care unit<br>- Other hospital unit<br>- Emergency department only	Yes	-
Date of admission	Yes	-
Sex<br>- Male<br>- Female<br>- Other	Yes	-
Ethnicity<br>- Non-Hispanic white<br>- Hispanic white<br>- Black<br>- Asian<br>- Other	Yes	-
Date of birth	Yes	-
Weight – kg	No	-
Height – cm	No	-
**Risk factors**		
Current cigarette smoking (Yes/No)	Yes	Consumption of ≥1 cigarette per day over the last year
Arterial hypertension (Yes/No)	Yes	Blood pressure of ≥140/90 mmHg at least twice before stroke or already under treatment with antihypertensive drugs
Dyslipidemia (Yes/No)	Yes	History of total blood cholesterol levels >220 mg/dL and/or total triglycerides levels >130 mg/dL and/or current used lipid-lowering drugs
Diabetes (Yes/No)	Yes	History of fasting glucose >126 mg/dL or the current use of hypoglycemic medications
Ischemic heart disease (Yes/No)	Yes	History of myocardial infarction, angina or prior evidence of coronary disease on coronary angiography
Chronic congestive heart failure (Yes/No)	Yes	History of stage C (structural heart disease and current or past history of heart-failure symptoms) or stage D (refractory symptoms that interfere with daily life or recurrent hospitalization despite targeted guideline-directed medical therapy) chronic heart failure
Chronic kidney disease (Yes/No)	Yes	History of estimated creatinine clearance of less than 60 for 3 months or more (including dialysis)

© 2026 D'Anna L et al. *JAMA Network Open*.

Chronic liver failure (Yes/No)	Yes	History of cirrhosis or end-stage liver disease
Symptomatic peripheral artery disease (Yes/No)	Yes	History of intermittent claudication of presumed atherosclerotic origin
Prior ischemic stroke or TIA (Yes/No)	Yes	-
Prior intracranial hemorrhage (Yes/No)	Yes	-
History of malignancy (Yes/No)	Yes	-
Type of malignancy<br>- Active<br>- In remission	Yes	We defined active malignancy as (1) a diagnosis of cancer that occurred within 6 months of the index event or during hospitalization, (2) cancer treatment with radiotherapy, chemotherapy or surgery or a combination of them within 6 months of the index event, (3) a previous history of malignancy and a diagnosis of recurrence or metastasis within 6 months of the index event. In remission was defined as a previous history of malignancy in the absence of active cancer criteria.
Site of malignancy<br>- Gastrointestinal<br>- Lung<br>- Genitourinary<br>- Breast<br>- Hematological<br>- Skin<br>- Other	Yes	-
Metastatic malignancy? (Yes/No)	Yes	-
Metastasis location<br>- Lymph nodes<br>- Liver<br>- Lung<br>- Bones<br>- Brain/spine/meningeal<br>- Other	Yes	-
Atrial fibrillation type<br>- Paroxysmal<br>- Persistent<br>- Long-standing persistent<br>- Permanent	Yes	According to the ACC/AHA/HRS guidelines classification
History of valvular heart disease (multiple choice)<br>- Sever mitral stenosis<br>- Sever aortic stenosis<br>- Severe mitral insufficiency<br>- Severe aortic insufficiency<br>- Mechanical heart valve<br>- Biological heart valve	Yes	-

© 2026 D'Anna L et al. *JAMA Network Open*.

Pacemaker (Yes/No)	Yes	-
Left atrial volume index at transthoracic echocardiography – mL/m^2	No	-
Left ventricle end-diastolic volume at transthoracic echocardiography – mL	No	-
Left ventricle end-systolic volume at transthoracic echocardiography – mL	No	-
Left ventricle ejection fraction at transthoracic echocardiography – mL	No	-
**Drugs history**		
Type of oral anticoagulant ongoing at the time of the index ischemic stroke<br>- Vitamin K antagonist<br>- Direct oral anticoagulant	Yes	-
Type of direct oral anticoagulant<br>- Apixaban<br>- Rivaroxaban<br>- Edoxaban<br>- Dabigatran	Yes	-
Type of vitamin K antagonist<br>- Warfarin<br>- Acenocoumarol<br>- Other	Yes	-
Time from last direct oral anticoagulant intake to admission<br>- <12 hours<br>- 12-24 hours<br>- 24-48 hours	Yes	-
Direct oral anticoagulant plasma levels or anti-factor Xa activity available on admission	Yes	-
Direct oral anticoagulant plasma levels (ng/mL) or anti-factor Xa activity on admission	Yes	-
Direct oral anticoagulant plasma levels or anti-factor Xa activity on admission<br>- Below range<br>- Within range<br>- Above range	Yes	In respect to the range locally determined for therapeutic anticoagulation
Direct oral anticoagulant at reduced dose at the time of the index ischemic stroke (Yes/No)	Yes	Apixaban 2.5mg BID, dabigatran 75mg BID, edoxaban 30mg daily, rivaroxaban 15mg daily

© 2026 D'Anna L et al. *JAMA Network Open*.

Direct oral anticoagulant reduced dose appropriate (Yes/No)	Yes	Reduced dose of apixaban was considered appropriate if 2 of 3 factors were present: 1) Age ≥80 years; 2) serum creatinine ≥1.5 mg/dL; 3) Weight ≤60 kg OR if apixaban is co-administered with combined P-gp and strong CYP3A4 inhibitors (e.g., ketoconazole, itraconazole, ritonavir). Reduced dose of dabigatran was considered appropriate if creatinine clearence 15-30 mL/min OR, creatinine clearence 30-50 mL/min with concomitant dronedarone or ketoconazole. Reduced dose of edoxaban was considered appropriate if creatinine clearence 15-50 mL/min. Reduced dose of rivaroxaban was considered appropriate if creatinine clearence ≤50 mL/min
Antihypertensive drugs on admission (Yes/No)	Yes	-
Lipid-lowering drugs on admission (Yes/No)	Yes	-
Antidiabetic drugs on admission (Yes/No)	Yes	-
Rhythm control drugs on admission (Yes/No)	Yes	-
Type of rhythm control drugs on admission<br>- Class I – Sodium channel blockers<br>- Class II – Beta-blockers<br>- Class III – Potassium channel blockers<br>- Class IV – Calcium channel blockers<br>- Class V – Miscellaneous agents (i.e., digoxin, ivabradine, adenosine)	Yes	According to the Vaughan Williams classification of antiarrhythmic drugs
Drugs potentially interfering with oral anticoagulation at the time of the index stroke (multiple choice)*<br>- Itraconazole<br>- Ketoconazole<br>- Clarithromycin<br>- Lopinavir<br>- Indinavir<br>- Ritonavir<br>- Telaprevir<br>- Voriconazole<br>- Any H2 inhibitor (i.e., cimetidine)<br>- Any proton pump inhibitor (i.e. omeprazole, pantoprazole)<br>- Doxorubicin<br>- Vinblastine<br>- Carbamazepine/ Oxcarbazepine<br>- Phenytoin<br>- Phenobarbital<br>- Rifampin<br>- Levetiracetam<br>- Valproic acid	No	-

© 2026 D'Anna L et al. *JAMA Network Open*.

- Dexamethasone<br>- Tocilizumab		
Antiplatelet therapy on admission (Yes/No)	Yes	-
Type of antiplatelet therapy on admission (multiple choice)<br>- Aspirin<br>- Clopidogrel<br>- Ticagrelor<br>- Ticlopidine<br>- Other	Yes	-
Antihypertensive drugs on admission (Yes/No)	Yes	-
**Clinical characteristics**		
Modified Rankin Scale (mRS) score before the index ischemic stroke	Yes	-
Type of ischemic stroke onset<br>- Known onset<br>- Wake-up stroke<br>- Unwitnessed stroke	Yes	
National Institute of Health Stroke Scale (NIHSS) score on admission (prior to any acute reperfusion therapy)	Yes	-
Acute ischemic stroke symptoms (multiple choice)<br>- Motor weakness<br>- Aphasia<br>- Dysarthria<br>- Sensory defect<br>- Visual field defect<br>- Diplopia<br>- Vertigo<br>- Loss of balance<br>- Hemineglect<br>- Other	No	-
Clinical classification of the acute ischemic stroke<br>- Total anterior circulation stroke (TACS)<br>- Partial anterior circulation stroke (PACS)<br>- Posterior circulation syndrome (PoCS)<br>- Lacunar stroke (LACS)	Yes	According to the Oxfordshire Stroke Project Classification
Admission systolic blood pressure - mmHg	No	-
Admission diastolic blood pressure – mmHg	No	-
Admission Glasgow Coma Scale (GCS) score	No	-

© 2026 D’Anna L et al. *JAMA Network Open*.

Presence of competing stroke etiology other than cardioembolism (Yes/No)	Yes	-
Type of competing stroke etiology other than cardioembolism<br>- Lacunar<br>- Large artery atherosclerosis<br>- Other determined etiology	Yes	According to the Trial of Org 10172 in the Acute Stroke Treatment (TOAST) classification system
Type of other determined etiology (specify)	Yes	-
Competing cardioembolic mechanisms other than atrial fibrillation (Yes/No)	Yes	-
Type of competing cardioembolic mechanisms other than atrial fibrillation (multiple choice)<br>- Reduced left ventricle ejection fraction (30-40%)<br>- Severely reduced left ventricle ejection fraction (< 30%)<br>- Left ventricle thrombus<br>- Endocarditis<br>- Mechanical heart valve<br>- Biological heart valve<br>- Atrial myxoma<br>- Fibroelastoma<br>- Other cardiac tumors<br>- Patent foramen ovale (PFO)<br>- Interventricular defect (IVD)<br>- Left ventricular aneurysm<br>- Left atrial aneurysm<br>- Other cardiac congenital alterations	Yes	-
Intravenous thrombolysis (Yes/No)	Yes	-
Endovascular thrombectomy (Yes/No)	Yes	-
Onset-to-needle time – minutes	Yes	-
Onset-to-groin time – minutes	Yes	-
Hemorrhagic infarction<br>- Yes - asymptomatic<br>- Yes – symptomatic<br>- No	Yes	Symptomatic hemorrhagic infarction was defined according to SITS-MOST as a neurologic deterioration of ≥4 NIHSS points or leading to death within 24 hours
Hemorrhagic infarction type (multiple choice)<br>- HI1: Scattered small petechiae, no mass effect<br>- HI2: Confluent petechiae, no mass effect<br>- PH1: Hematoma within infarcted tissue, occupying < 30%, no substantive mass effect	Yes	According to the Heidelberg classification system

© 2026 D'Anna L et al. *JAMA Network Open*.

- PH2: Hematoma occupying 30% or more of the infarcted tissue, with obvious mass effect PH remote from infarcted brain tissue<br>- rPH: Remote parenchymal hematoma<br>- Intraventricular hemorrhage<br>- Subarachnoid hemorrhage<br>- Subdural hemorrhage		
National Institute of Health Stroke Scale (NIHSS) score at 24 hours	No	-
**Neuroimaging information**		
Type of brain neuroimaging performed on admission<br>- Non-contrast computed tomography<br>- Magnetic resonance imaging<br>- Both	Yes	-
Type of brain neuroimaging performed at 24 hours follow-up<br>- Non-contrast computed tomography<br>- Magnetic resonance imaging<br>- Both<br>- None	Yes	-
Type of brain vessel imaging performed (multiple choice)<br>- Computed tomography angiography - extracranial vessels<br>- Computed tomography angiography - intracranial vessels<br>- Magnetic resonance angiography - extracranial vessels<br>- Magnetic resonance angiography - intracranial vessels<br>- Color Doppler ultrasonography - extracranial vessels<br>- Color Doppler ultrasonography - intracranial vessels<br>- X-ray angiography<br>- None	Yes	-
Large vessel occlusion (Yes/No/Unknown)	Yes	-
Large vessel occlusion site (multiple choice)<br>- Middle cerebral artery - M1<br>- Middle cerebral artery - M2<br>- Middle cerebral artery - More distal than M2<br>- Tandem occlusion<br>- Anterior cerebral artery<br>- Posterior cerebral artery	Yes	-

© 2026 D'Anna L et al. *JAMA Network Open*.

- Basilar artery<br>- Internal carotid artery<br>- Vertebral artery		
Baseline modified Thrombolysis in Cerebral Infarction score<br>- Grade 0: no perfusion<br>- Grade 1: antegrade reperfusion past the initial occlusion, but limited distal branch filling with little or slow distal reperfusion<br>- Grade 2a: antegrade reperfusion of less than half of the occluded target artery previously ischemic territory (i.e., in one major division of the middle cerebral artery and its territory)<br>- Grade 2b: antegrade reperfusion of more than half of the previously occluded target artery ischemic territory (i.e., in two major divisions of the MCA and their territories)<br>- Grade 2c: near complete perfusion except for slow flow or distal emboli in a few distal cortical vessels<br>- Grade 3: complete antegrade reperfusion of the previously occluded target artery ischemic territory, with absence of visualized occlusion in all distal branches	Yes	-
Post-endovascular thrombectomy modified Thrombolysis in Cerebral Infarction score<br>- Grade 0: no perfusion<br>- Grade 1: antegrade reperfusion past the initial occlusion, but limited distal branch filling with little or slow distal reperfusion<br>- Grade 2a: antegrade reperfusion of less than half of the occluded target artery previously ischemic territory (i.e., in one major division of the middle cerebral artery and its territory)<br>- Grade 2b: antegrade reperfusion of more than half of the previously occluded target artery ischemic territory (i.e., in two major divisions of the MCA and their territories)<br>- Grade 2c: near complete perfusion except for slow flow or distal emboli in a few distal cortical vessels<br>- Grade 3: complete antegrade reperfusion of the previously occluded target artery ischemic territory,	Yes	-

© 2026 D’Anna L et al. *JAMA Network Open*.

with absence of visualized occlusion in all distal branches		
Presence of extracranial internal artery stenosis ≥50% ipsilateral to the acute ischemic stroke lesion (Yes/No)	Yes	-
Degree of extracranial internal artery stenosis ≥50% ipsilateral to the acute ischemic stroke lesion<br>- 50-69%<br>- 70-79%<br>- 80-99%<br>- Occlusion (100%)	Yes	-
Presence of extracranial vertebral artery stenosis ≥50% ipsilateral to the acute ischemic stroke lesion (Yes/No)	Yes	-
Degree of extracranial vertebral artery stenosis ≥50% ipsilateral to the acute ischemic stroke lesion<br>- 50-69%<br>- 70-79%<br>- 80-99%<br>- Occlusion (100%)	Yes	-
Presence of intracranial artery stenosis (Yes/No)	Yes	-
Site of intracranial artery stenosis (multiple choice)<br>- Middle cerebral artery<br>- Anterior cerebral artery<br>- Posterior cerebral artery<br>- Basilar artery<br>- Internal carotid artery<br>- Vertebral artery	Yes	-
Presence of symptomatic intracranial artery stenosis (Yes/No)	Yes	-
**Laboratory tests**		
International Normalized Ratio (INR)	Yes	-
aPTT – seconds	No	-
PT – seconds	No	-
Red blood cells – millions/mm^3	No	-
White blood cells – millions/mm^3	No	-
Lymphocytes – millions/mm^3	No	-
Neutrophiles – millions/mm^3	No	-
Platelets – thousands/mm^3	No	-
Hemoglobin – mg/dL	No	-
Admission blood glucose levels – mg/dL	No	-

© 2026 D'Anna L et al. *JAMA Network Open*.

Fasting blood glucose levels – mg/dL	No	-
C reactive protein – mg/dL	No	-
Creatinine – mg/dL	No	-
Creatinine clearance – mL/min	No	-
Glycate hemoglobin – mmol/L	No	-
Total cholesterol – mg/dL	No	-
High density lipoprotein (HDL) – mg/dL	No	-
Low density lipoprotein (LDL) – mg/dL	No	-
Triglycerides – mg/dL	No	-
ALT/GPT – U/L	No	-
AST/GOT – U/L	No	-
Gamma-GT – U/L	No	-
Total bilirubin – mg/dL	No	-

* *Collection of concomitant drugs potentially interacting with oral anticoagulation was not mandatory in the ASPERA-R registry and may be incomplete across participating sites; data are therefore reported as available cases. The list reflects the prespecified baseline variables collected in the registry. For the purpose of the present analysis, we included drugs as having established or potential/uncertain interactions based on Summary of Product Characteristics (EMA) and established pharmacokinetic mechanisms (eg, CYP3A4 and/or P-glycoprotein modulation). These variables were not used to define exposure groups or included in the primary adjustment model and were collected to enable exploratory and secondary analyses, including analyses planned for a separate dedicated report[1–4].*

1. **Boehringer Ingelheim. Pradaxa 150 mg hard capsules - Summary of Product Characteristics (SmPC) - (emc). *https://www.medicines.org.uk/emc/product/4703/smpc*. 2022;**
2. **Daiichi Sankyo UK Limited. Lixiana 30mg Film-Coated Tablets - Summary of Product Characteristics (SmPC) - (emc). *https://www.medicines.org.uk/emc/product/6906/smpc*. 2022;**
3. **Bristol-Myers Squibb-Pfizer. Eliquis 2.5 mg film-coated tablets - Summary of Product Characteristics (SmPC) - (emc). *https://www.medicines.org.uk/emc/product/4756/smpc*. 2022;**
4. **Bayer. Xarelto 20mg film-coated tablets - Summary of Product Characteristics (SmPC) - (emc). *https://www.medicines.org.uk/emc/product/2793/smpc#*. 2022;**

© 2026 D'Anna L et al. *JAMA Network Open*.

eAppendix 3. Protocol Synopsis

Continuation versus Switching of Direct Oral Anticoagulant Therapy After Breakthrough Ischemic Stroke in Atrial Fibrillation.

Emulated Target Trial within the ASPERA-R Registry

**Background and Rationale**	Breakthrough ischemic stroke in patients receiving direct oral anticoagulants (DOACs) for atrial fibrillation (AF) represents a major therapeutic challenge. In clinical practice, many clinicians switch anticoagulant therapy after such events, despite limited comparative evidence supporting superiority of switching over continuation. Given the absence of randomized trials addressing this question, we designed a target trial emulation within the ASPERA-R registry to compare continuation versus switching of oral anticoagulation using a prespecified non-inferiority framework.
**Study Objective**	To determine whether continuation of the same DOAC after a breakthrough ischemic stroke is non-inferior to switching oral anticoagulant therapy with respect to 90-day net clinical benefit.
**Trial Design (Target Trial Emulation Framework)**	This study emulates a pragmatic, parallel-group, non-inferiority randomized controlled trial.<br>• Design: Multicenter observational cohort with causal inference framework<br>• Allocation: Observed treatment strategy (continuation vs switching)<br>• Analysis population: Eligible registry patients meeting prespecified criteria<br>• Primary estimand: Marginal (population-average) treatment effect<br><br>All components of the target trial were prespecified before database lock.
**Study Setting**	ASPERA-R (Retrospective arm of ASPERA registry)<br>35 stroke centers across 9 countries (Europe and North Africa)<br><br>Participating centers were required to have:<br>• Standardized etiological stroke work-up<br>• Stroke unit–based management<br>• Structured 90-day follow-up pathway<br>• Proactive outcome ascertainment (including patient contact if scheduled visits were missed)
**Eligibility Criteria (Prespecified)**	**Inclusion Criteria**<br>1. Age ≥18 years<br>2. Acute ischemic stroke confirmed by neuroimaging<br>3. Documented atrial fibrillation

© 2026 D’Anna L et al. *JAMA Network Open*.

	4. Continuous DOAC intake in the 7 days preceding stroke<br>5. Last DOAC dose ≤48 hours before stroke<br>6. Resumption of oral anticoagulant therapy (DOAC or VKA) after index event<br>7. Available 90 ±10 day follow-up<br><br>**Exclusion Criteria**<br>1. Mechanical prosthetic heart valve<br>2. Pre-stroke treatment with VKA or heparin<br>3. Transition to heparin/LMWH as post-stroke anticoagulation strategy<br>4. Inappropriate DOAC dosing per guideline criteria<br>5. Documented non-adherence<br>6. Left atrial appendage occlusion<br>7. Missing mandatory baseline covariates<br><br>All criteria were defined before statistical analysis.
**Treatment Strategies**	**Strategy A (Reference): DOACSAME**<br><br>Continuation of the same DOAC used prior to the index stroke.<br><br>**Strategy B (Intervention): DOACSWITCH**<br><br>Switch to an alternative oral anticoagulant after index stroke:<br>• Switch to another DOAC (same or different mechanism), or<br>• Switch to vitamin K antagonist (VKA)<br><br>Treatment strategy classification was based on anticoagulant resumed after the index stroke
**Time zero**	Time zero was defined as the date of the index ischemic stroke.Baseline covariates were measured at or prior to time zero to avoid immortal time bias.
**Follow-up**	Participants were followed from time zero to 90 days (±10 days).<br><br>Outcome ascertainment was standardized:<br>• Structured in-person visit when possible<br>• Direct patient contact if needed<br>• Local adjudication using predefined outcome definitions
**Outcomes (Prespecified)**	**Primary Outcome**<br><br>Net Clinical Benefit (NCB) at 90 days, defined as:<br>• Recurrent ischemic stroke, or<br>• Moderate-to-severe bleeding (GUSTO criteria)<br><br>**Secondary Outcomes**<br>• Recurrent ischemic stroke<br>• Any ischemic event (stroke, TIA, systemic embolism)

© 2026 D'Anna L et al. *JAMA Network Open*.

	• Symptomatic intracranial hemorrhage (sICH)<br>• Moderate-to-severe bleeding<br>• All-cause mortality<br>• Vascular mortality<br><br>All definitions were prespecified and uniformly applied.
**Non-Inferiority Framework**	The primary hypothesis tested whether continuation (DOACSAME) was non-inferior to switching (DOACSWITCH).<br><br>**Primary Effect Measure**<br><br>Absolute risk difference (RD)<br><br>Risk differences expressed as:<br>DOACSWITCH – DOACSAME<br><br>**Prespecified Non-Inferiority Margins**<br>• +3.0% for primary composite endpoint<br>• +0.5% for sICH<br>• +1.0% for moderate-to-severe extracranial bleeding<br><br>Margins were defined a priori based on prior secondary prevention trials and regulatory guidance.<br><br>**Statistical Inference**<br>• Two-sided 90% confidence intervals<br>• Equivalent to one-sided $\alpha = 0.05$<br>• Non-inferiority concluded if the upper bound of the 90% CI did not exceed the prespecified margin
**Sample Size Considerations (Prespecified Planning Assumptions)**	Before conducting the target trial emulation, the following planning assumptions were defined:<br>• Expected event rate in DOACSAME group: ~5%<br>• Non-inferiority margin: 3% (absolute RD)<br>• One-sided $\alpha = 0.05$<br><br>Under these assumptions, approximately 800 patients (400 per group) would provide 80% power to exclude a clinically unacceptable excess risk.<br><br>The final cohort exceeded this threshold.
**Confounding Control Strategy**	To emulate randomization:<br>• Propensity score–based inverse probability of treatment weighting (IPTW)<br>• Stabilized weights<br>• Covariates selected a priori<br>• Balance assessed using standardized mean differences (SMD <0.1 threshold)

© 2026 D’Anna L et al. *JAMA Network Open*.

	**Prespecified Sensitivity Analyses**<br>• IPTW trimming<br>• Overlap weighting<br>• Augmented IPTW (AIPW)<br>• Per-protocol analysis (resumption ≤10 days)<br>• Exclusion of competing stroke etiologies
**Ethical Considerations**	Approved by the territorial ethics committee of the Abruzzo region (CEtRA), approval code 033054/25.

© 2026 D'Anna L et al. *JAMA Network Open*.
