# Supplementary material for: Continuation vs Switching Direct Oral Anticoagulant Therapy After Breakthrough Stroke
Source: JAMA Netw Open. 2026 Apr 28;9(4):e269584. doi: 10.1001/jamanetworkopen.2026.9584 (PMC13126222; doi:10.1001/jamanetworkopen.2026.9584)
Supplement: Supplement 2. — Data Sharing Statement [file jamanetwopen-e269584-s002.pdf]

Data Sharing Statement

D’Anna. Continuation vs Switching Direct Oral Anticoagulant Therapy After Breakthrough Stroke. *JAMA Netw Open*. Published April 28, 2026. doi:10.1001/jamanetworkopen.2026.9584

Data
Data available: No
